# Mapping Interactions of Microbial Metabolites with Human G-Protein-Coupled Receptors

**DOI:** 10.1016/j.chom.2019.07.002

**Published:** 2019-08-14

**Authors:** Dominic A. Colosimo, Jeffrey A. Kohn, Peter M. Luo, Frank J. Piscotta, Sun M. Han, Amanda J. Pickard, Arka Rao, Justin R. Cross, Louis J. Cohen, Sean F. Brady

**Affiliations:** 1 Laboratory of Genetically Encoded Small Molecules, the Rockefeller University, 1230 York Avenue, New York City, NY 10065, USA; 2 Donald B. and Catherine C. Marron Cancer Metabolism Center, Memorial Sloan Kettering Cancer Center, New York City, NY 10065, USA; 3 Division of Gastroenterology, Department of Medicine, Icahn School of Medicine at Mount Sinai, New York City, NY 10029, USA

**Keywords:** primary metabolites, human microbiome, G protein-coupled receptors

## Abstract

Despite evidence linking the human microbiome to health and disease, how the microbiota affects human physiology remains largely unknown. Microbiota-encoded metabolites are expected to play an integral role in human health. Therefore, assigning function to these metabolites is critical to understanding these complex interactions and developing microbiota-inspired therapies. Here, we use large-scale functional screening of molecules produced by individual members of a simplified human microbiota to identify bacterial metabolites that agonize G-protein-coupled receptors (GPCRs). Multiple metabolites, including phenylpropanoic acid, cadaverine, 9-10-methylenehexadecanoic acid, and 12-methyltetradecanoic acid, were found to interact with GPCRs associated with diverse functions within the nervous and immune systems, among others. Collectively, these metabolite-receptor pairs indicate that diverse aspects of human health are potentially modulated by structurally simple metabolites arising from primary bacterial metabolism.

## Highlights

• Metabolite library from human microbiota screened for direct agonism of 241 GPCRs
• Taxa-specific primary metabolites agonize individual GPCRs or broad GPCR families
• Bacteria agonize receptors linked to metabolism, neurotransmission, and immunity
• Simple bacterial metabolites may play a role in modulating host pathways

## Graphical Abstract

**Figure:**
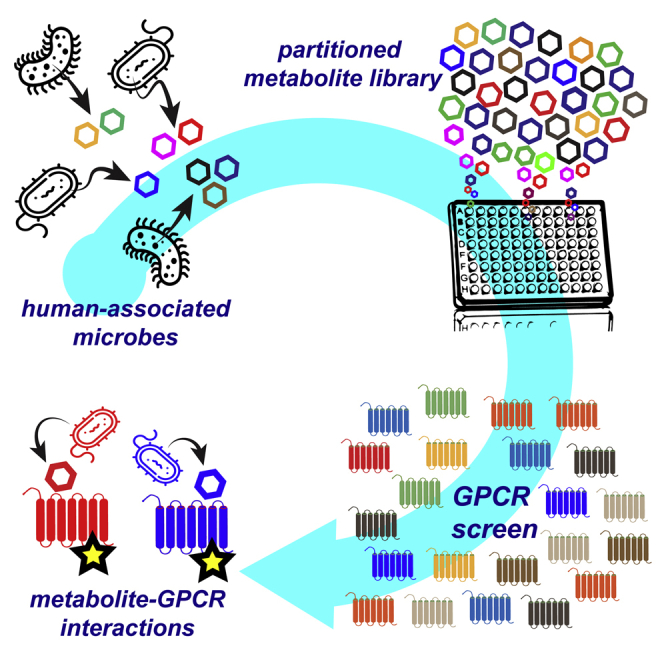

## Teaser

Colosimo et al. use functional screening of small molecules produced by individual members of a simplified human microbiota to identify bacterial metabolites that agonize G protein-coupled receptors (GPCRs). These results indicate that diverse aspects of human health are potentially modulated by structurally simple metabolites arising from primary bacterial metabolism.

## Introduction

Human bodies are home to diverse and ever-changing collections of bacteria. The ability of the microbiota to influence human health has been explored extensively (Knight et al., 2017). In addition to hypothesis-driven studies in model organisms, one of the most common methods for studying host-microbe interactions has featured “omics”-based analyses that have examined genomic, transcriptomic, proteomic, or metabolic differences between patient cohorts (Fritz et al., 2013, Gagliani et al., 2014, Hugenholtz and de Vos, 2018, Qin et al., 2012, Qin et al., 2014, Turnbaugh et al., 2009). Although these informatics-based methods have served as powerful tools for uncovering correlations between changes in the microbiota and health and disease, they are somewhat limited in their ability to reveal the mechanistic details of how the microbiota might alter mammalian physiology (Cani, 2018). Much of the influence the microbiota has on its human host is likely encoded in the collection of small molecules it produces or modulates (Brown and Hazen, 2017). The number of well-defined interactions between metabolites produced by human associated bacteria and discrete human receptors is dwarfed by the number of reports attributing biological phenotypes to the microbiome, highlighting the need for a more systematic characterization of microbiota-encoded bioactive metabolites.

In the case of synthetic small molecules that have proved useful for therapeutically modulating human physiology (i.e., U.S. Food and Drug Administration (FDA) approved drugs) the majority (60%–70%) function through just three classes of receptors: G-protein-coupled receptors (GPCR), ion channels, or nuclear hormone receptors (Santos et al., 2017). Many of these same proteins bind endogenous signaling molecules that regulate a wide range of physiological responses (Rosenbaum et al., 2009). Based on the fact that these receptors play such an important role in how eukaryotic cells have evolved to translate external chemicals into biologic responses, it is likely that the microbiota affects host physiology by modulating these same receptors with secreted metabolites.

Although healthy humans are colonized by hundreds, if not thousands, of different bacterial species, the metabolic diversity they generate is likely limited by a high level of biosynthetic redundancy between bacterial species (Dorrestein et al., 2014). Partly because of this metabolic redundancy, it has been possible to use simplified human microbiomes (SIHUMIs) to model health and disease in murine models (Kovatcheva-Datchary et al., 2019, Subramanian et al., 2014). In lieu of exploring random individual commensal species, we sought to conduct a more in-depth investigation of GPCR-active microbiota-encoded metabolites using bacteria from a model SIHUMI that contained a taxonomically diverse collection of commensal, health promoting, and pathogenic bacteria. This consortium, which is composed of seven bacteria, assembled as a tool for studying gastrointestinal (GI) inflammation in the context of a healthy bacterial flora fulfills these general criteria and was therefore selected for use in this study (Eun et al., 2014). Bacteria present in this SIHUMI consortium include beneficial bacteria (*Lactobacillus plantarum*, *Bifidobacterium longum*, and *Faecalibacterium prauznitzii*), non-pathogenic bacteria associated with disease (*Bacteroides vulgatus* and *Ruminococcus gnavus*), and clinically relevant pathogens (*Escherichia coli* LF-82 and *Enterococcus faecalis*).

We screened the metabolites produced by individually grown members of this SIHUMI consortium for agonism against 241 GPCRs. The resulting interaction map provides evidence, at the molecular level, for the existence of multiple potential microbiota metabolite-host interactions, many of which involve receptors that have been modulated therapeutically with synthetic small molecules. Our characterization of interactions predicted by this analysis led to the discovery of both previously unrecognized as well as known microbiota-encoded GPCR agonists. The structures of the active molecules we identified support the growing notion that simple bacterial metabolites arising from primary metabolic processes are likely to broadly impact human physiology.

## Results

### Culturing Bacteria and GPCR Screening

**Figure 1:**
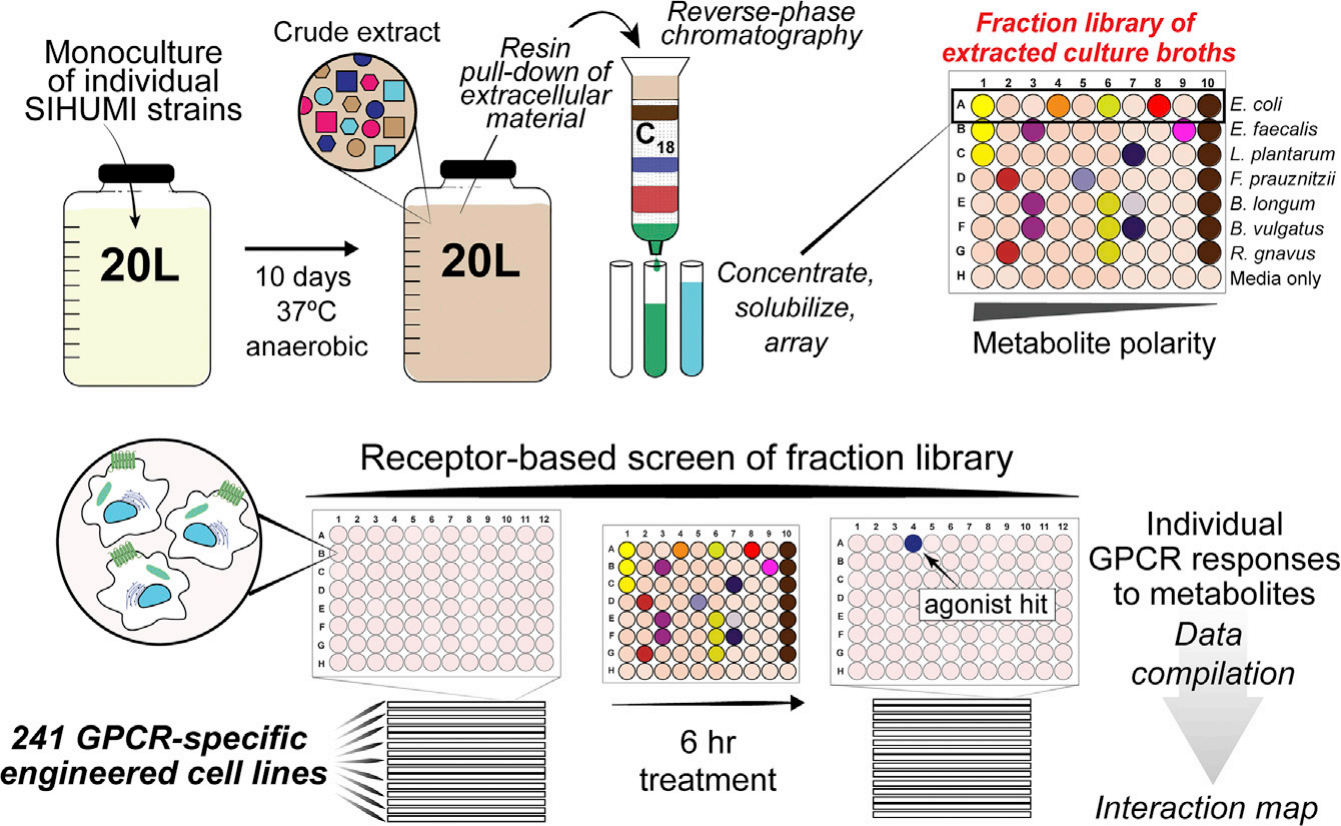
Experimental Procedure for Generating and Screening Library of Secreted Bacterial Metabolites from Large-Scale Monocultures of SIHUMI Consortium Members This library was screened for the ability to agonize 241 distinct GPCRs.

Bacteria from the SIHUMI consortium were individually fermented under anaerobic conditions in separate large-scale (20 L) culture vessels (Figure 1). After 10 days of static fermentation at 37°C, hydrophobic resin was added directly to each culture. The resulting suspension was mixed to allow organic metabolites present in the fermentation broth to bind to the absorbent resin. Metabolite loaded resin was then collected by filtration, washed, and the bound metabolites were eluted with acetone. Each resulting crude metabolite extract was partitioned into 9 metabolite-rich fractions using reversed-phase flash chromatography. A small aliquot of each fraction, alongside an aliquot of the original crude extract, was arrayed for use in high-throughput GPCR screening. The remaining material was saved for follow-up assays and for use in molecule isolation and structure elucidation studies. Although this pre-fractionation process increases the number of samples to be screened, it simplifies the complexity of the crude culture broth extracts, which should improve the signal in the primary screen thereby increasing the diversity of interactions that are identified and facilitating the downstream isolation of bioactive compounds (Butler et al., 2014, Wagenaar, 2008). In addition to the bacterial fermentations, media not inoculated with bacteria were processed under identical conditions to control for the possible bioactivity of small molecules derived directly from the media. The resulting library of bacterial metabolites was then screened with a cell-based assay for fractions that could agonize members of a panel of 241 GPCRs (Table S1). Specifically, a collection of recombinant cell lines engineered to measure β-arrestin recruitment by individual GPCR targets (β-arrestin recruitment assay) was used. For GPCRs with well-characterized endogenous ligands, a maximum value for β-arrestin recruitment (100%) was set by exposing the recombinant cell line to a known agonist (Table S1). In the case of orphan receptors (i.e., receptors without well-characterized endogenous ligands), β-arrestin recruitment was normalized relative to the vehicle control by assigning a 2-fold increase in raw luminescence as 100% activity. Hits were classified as such if a fraction induced a GPCR response to >30% of the control ligand (>50% for orphan GPCRs) and the comparable media control fraction showed <30% activity against the same GPCR (<50% for orphan GPCRs).

**Figure 2:**
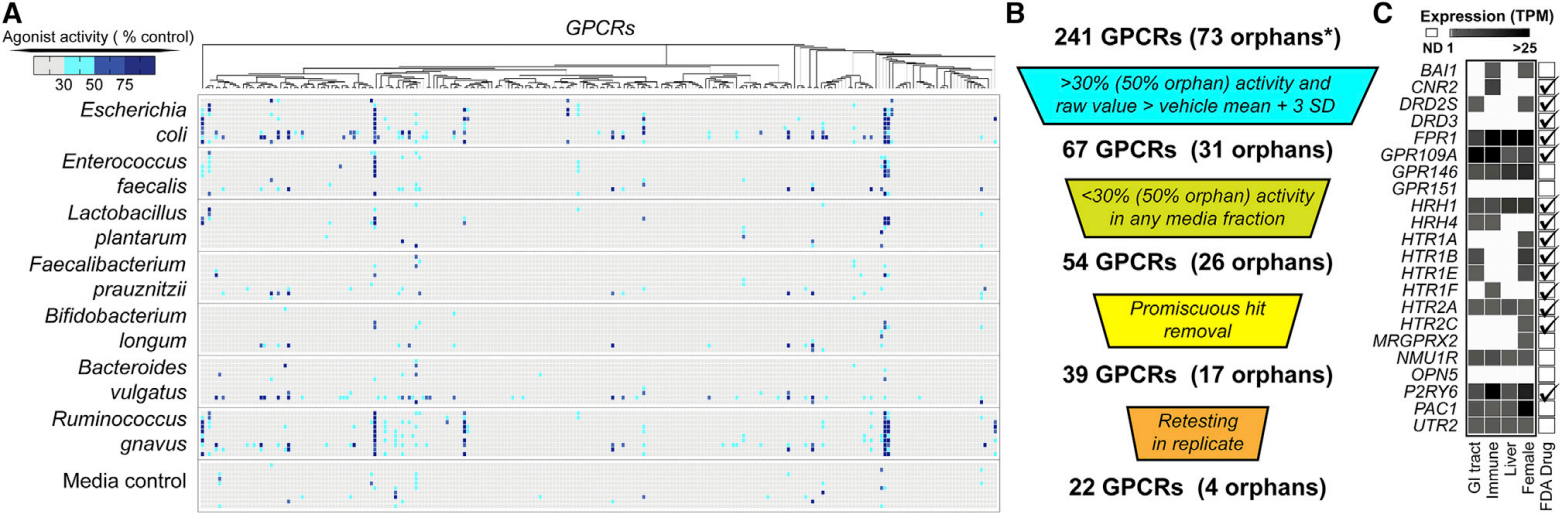
Overview of GPCR Screening Results (A) Heatmap of individual assays for each GPCR tested, indicating *β*-arrestin recruitment response normalized to endogenous or synthetic control compound (100%). For each bacterial strain, the 9 fractions are vertically displayed followed by the crude extract of that strain. (B) GPCR hit prioritization scheme. (C) Subset of GPCRs that show <30% (50% for orphans) response to the media control but have >30% response (50% for orphans) to a bacterial fraction. The orphan receptors in this pool are BAI1, GPR146, GPR151, and OPN5. Receptor gene expression levels in tissues commonly exposed to the human microbiome [Transcripts per Million (TPM)]. Data is from the Human Protein Atlas (Uhlén et al., 2015). Receptors targeted by approved FDA drugs are indicated on the right (Sriram and Insel, 2018).

The bacterial fraction library induced β-arrestin recruitment above our hit threshold levels for 67 of the 241 individual GPCR reporter cell lines we tested (Figures 2A and 2B; Table S2). Of these 67 GPCRs, 54 did not show a strong background signal from the corresponding media control fraction, suggesting they were responding to bacterially encoded metabolites. Manual review of these 54 hits led us to de-prioritize 15 of these GPCR-fraction pairs because of high background of either the receptor or fraction (Table S3). The remaining 39 GPCRs were re-assayed in replicate; 22 of these GPCRs showed reproducible β-arrestin recruitment in response to 1 or more bacterial fractions (Figure 2C). Of these 22 validated interactions, only 8 reached our hit threshold level in an identical GPCR screen using crude bacterial culture broth extracts (Figure S1), thus supporting our original hypothesis that pre-fractionation methods would enable the discovery of a larger number of GPCR interactions.

A large number of the receptors that were reproducibly agonized by microbiota-encoded metabolites are also targeted by FDA approved drugs, indicating that receptors with proven physiological relevance are potentially modulated by bacterial ligands (Figure 2C). Based on data from the Human Protein Atlas, most of the receptors that reproducibly responded to bacterial metabolites are expressed at body sites regularly exposed to the microbiota (Figure 2C) (Uhlén et al., 2015). We focused on the characterization of agonists for receptors with demonstrated expression in either the GI tract or in immune cells that infiltrate and survey the GI tract. In both cases, receptors would be activated as a result of metabolites accumulating in proximity to the GI epithelium and not require the metabolite to circulate peripherally in the bloodstream. To identify specific GPCR-active metabolites, we used bioassay-guided isolation to purify metabolites from the large-scale culture broth fractions and *de novo* structure elucidation methods to determine their structures.

### Bacterial Ligands for Hydroxycarboxylic Acid Receptors

**Figure 3:**
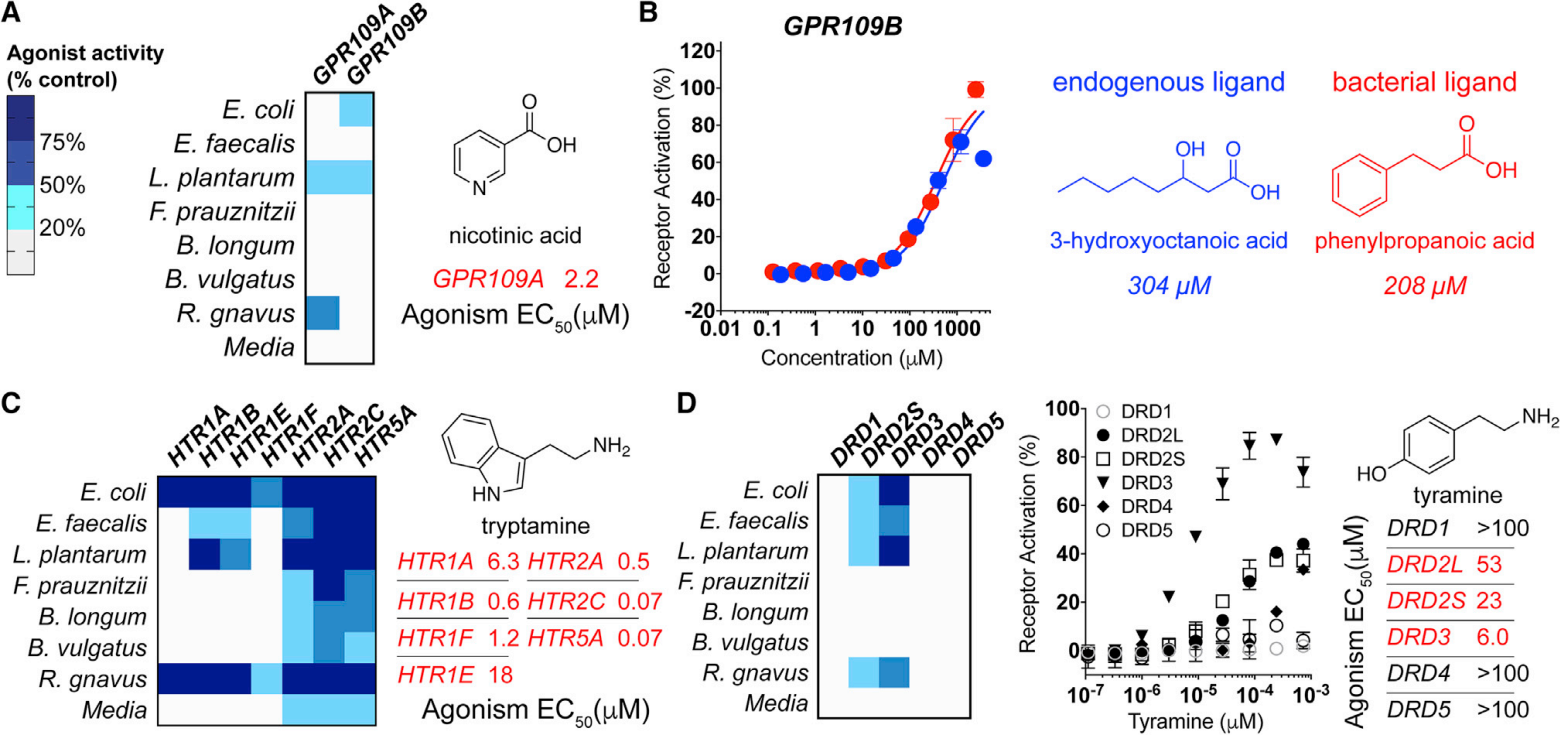
Bacterial Ligands for Hydroxycarboxylic Acid and Neurotransmitter Receptors The single fraction with maximum activity for each bacterial strain is depicted in heatmaps. (A) Left, heatmap depicting agonism of GPR109A and GPR109B by bacterial fractions. Right, agonist activity (EC_50_) of purified nicotinic acid against GPR109A. (B) Left, dose-response curves (DRCs) for known and previously unknown GPR109B agonists (right). (C) Left, heatmap depicting agonism of HTR receptors by culture broth extract fractions. Right, agonist activity (EC_50_) of tryptamine against HTRs. (D) Left, heatmap depicting agonism of DRD family receptors by culture broth extract fractions. Right, agonist activity (EC_50_) of tyramine against DRDs. All dose-response curves were run in duplicate. Error bars are standard deviation. Error bars that are shorter than the height of the symbol are not shown.

A number of receptors agonized in our screen are known to respond to bacterial ligands. As an initial validation exercise, we characterized activities expected to arise from well-known bacterial GPCR agonists. The hydroxycarboxylic acid receptors, GPR81, GPR109A, and GPR109B, are agonized by both human and bacterial ligands (Offermanns, 2017). Bioassay-guided fractionation of GPR109A active fractions from cultures of both *L*. *plantarum* and *R*. *gnavus* yielded nicotinic acid (vitamin B3) as the active metabolite (Figures 3A and S2). Nicotinic acid, an essential nutrient acquired either through diet or gut bacteria, is the most extensively studied non-endogenous ligand for this receptor. Its ability to regulate lipid metabolism in hyperlipidemic patients is well established in the clinic (Garg et al., 2017). The identification of this well characterized and *in vivo* validated ligand-receptor pair suggests that the data generated in our screen have the potential to uncover biologically relevant metabolite GPCR interactions.

Fractions derived from cultures of both *E*. *coli* LF82 and *L*. *plantarum* agonized a second hydroxycarboxylic acid receptor, GPR109B. Bioassay-guided fractionation did not identify the endogenous ligand produced in humans, 3-hydroxyoctanoic acid but instead yielded phenylpropanoic acid as the active metabolite (Figures 3B and S2). This previously unknown GPR109B agonist elicited a similar GPCR response to 3-hydroxyoctanoic acid (Figure 3B). While the EC_50_ values for the known and bacterial ligands (304 and 208 μM, respectively) are higher than is often seen for endogenous GPCR ligands (Table S1), no more potent GPR109B agonists have been identified outside of those derived synthetically (Jung et al., 2007). Whether this is an inherent attribute of the receptor or represents a failure to identify the natural human ligand for this receptor remains to be seen.

Phenylpropanoic acid is not produced by human eukaryotic cells. Its presence in human fecal and sera samples has been attributed to either *de novo* biosynthesis by bacteria or microbial transformation of dietary compounds, most notably by species of *Clostridium* (Gao et al., 2009, Loke et al., 2009; Muñoz-González et al., 2013, Rowland et al., 2018). While we demonstrate that phenylpropanoic acid is a microbiota-derived agonist of GPR109B, in a screen of synthetic molecules, several aromatic D-amino acids were found to be GPR109B agonists that can trigger chemoattraction signaling pathways in leukocytes (Ahmed et al., 2009, Irukayama-Tomobe et al., 2009). In a quantitative analysis of human fecal water, phenylpropanoic acid was reported in healthy patients at an average concentration of 77.30 μg/mL (513 μM) (Gao et al., 2009). At this concentration, production of phenylpropanoic acid by gut bacteria would be high enough to agonize GPR109B.

### Aromatic Amines Agonize Neurotransmitter Receptors

Our GPCR interaction map revealed numerous bacterial fractions that strongly agonized neurotransmitter receptors, a key component of the gut-brain axis (Figure 1C) (Mittal et al., 2017). Bacterially produced aromatic amines, most notably tryptamine, have recently been reported as agonists of neurotransmitter receptors, particularly serotonergic GPCRs (5-hydroxytryptamine receptors, or HTRs) (Bhattarai et al., 2018). A majority of bacteria in this SIHUMI produced fractions that agonized HTRs (Figure 3C). Isolation of the active metabolite yielded tryptamine, which was produced in varying quantities by members of this SIHUMI (Figure S3). These results agree with various reports that HTRs are responsive to a wide array of bacteria because of the generality of tryptamine production across species (Luqman et al., 2018).

In fractions from multiple bacterial species, we observed agonism of the D2-type dopamine receptors (DRDs), DRD2 and DRD3 (Figure 3D). Bioassay-guided isolation led to the aromatic amine tyramine as the major metabolite responsible for DRD agonism in these fractions (Figure S3). Tyramine arises from decarboxylation of tyrosine and differs from dopamine only by the absence of a second hydroxyl on the aromatic ring. It is reported to accumulate to μM levels in the GI tract, a phenomenon which has been attributed to production by human microbiota (Sridharan et al., 2014). While no biological significance has been assigned to the microbiota-dependent accumulation of tyramine in animal models, it is sufficiently potent that its observed concentration in the GI tract is high enough to agonize D2-subtype DRDs.

### Polyamine Ligand for a Histamine Receptor Family Member

**Figure 4:**
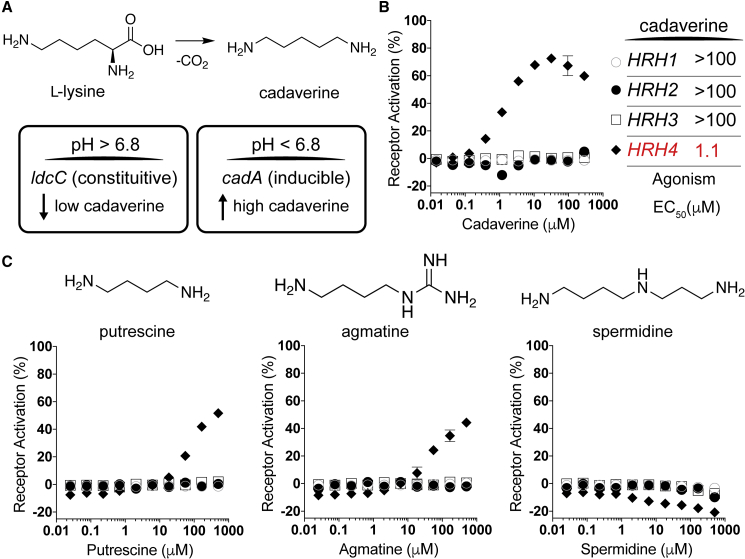
Cadaverine Is a Bacterial Ligand for a Specific Histamine Receptor (A) Top, schematic of cadaverine biosynthesis from L-lysine. Bottom, bacterial enzymes that catalyze this reaction include LydC, which is constitutively expressed and CadA, whose gene expression is induced at low pH. (B) Dose-response curves for cadaverine against HRH family receptors. (C) Dose-response curves (bottom) of bacterial polyamines (above) against HRH family receptors. Receptor symbols are labeled as in (B). All dose-response curves were run in duplicate. Error bars are standard deviation. Error bars that are shorter than the height of the symbol are not shown.

In contrast to the broad activation seen for DRDs and HTRs across extracts from all of the bacteria in this consortium, a specific response to fractions from *E*. *coli* LF82 was detected for a member of the histamine receptor (HRH) family, HRH4. Our inability to retain HRH4-activity when using hydrophobic chromatography during the bioassay-guided purification process suggested that the active molecule was highly polar. We did not, however, expect that the activity was due to bacterially produced histamine, as the active fraction did not agonize other HRH family receptors and we could not detect histamine by LC-MS or NMR. We ultimately found the polyamine cadaverine to be the metabolite responsible for HRH4 agonism (Figure 4A). The activity of cadaverine was confirmed using a commercial standard (EC_50_, 1.1 μM) (Figure 4B). In addition to cadaverine, bacteria commonly produce a number of other simple polyamines including agmatine, spermidine, and putrescine (Michael, 2018). To explore the promiscuity of HRH4 agonism by polyamines, we tested synthetic standards of these metabolites for the ability to induce β-arrestin recruitment by each member of the HRH receptor family. Agmatine and putrescine showed limited activity against HRH4 (Figure 4C), while spermidine did not show activity against any receptor in the family. The inability of humans to biosynthesize cadaverine suggests that the influence of cadaverine on histamine signaling pathways is likely specific to bacterial metabolism.

Cadaverine is produced by bacteria through the decarboxylation of lysine (Figure 4A), while agmatine and putrescine are derived from arginine. In a number of bacteria, including many *E*. *coli* strains and other species associated with the human microbiota (Table S4), cadaverine is encoded by both the constitutive *ldc* gene cluster as well as the *cad* gene cluster, which is induced at low pH (pH < 6.8) (Ma et al., 2017). High-level production of cadaverine by the CadA lysine decarboxylase is known to play a role in protecting against acid stress (Moreau, 2007). To confirm the presence of this acid-stress response in our *E*. *coli* LF82 strain, we constructed *cadA* knockout strains and observed *cadA*-dependent accumulation of extracellular cadaverine in response to growth media acidification (Figure S4). As the pH of the digestive system varies longitudinally and features multiple acidic sections (e.g., cecum pH ∼5.7), increased production of cadaverine by *cad* gene cluster containing bacteria is likely to occur at numerous sites in the GI tract. The biological relevance of GI production of polyamines remains unclear; however, host responses to polyamines have been reported in various contexts (Kovács et al., 2019, Paik Jung and Bjeldanes, 1979). Interestingly, although HRH subtypes differ in their associated functions and their distribution throughout the human body, HRH4 is expressed in the GI tract and altered expression levels have been linked to inflammatory responses that are related to inflammatory bowel diseases and cancer (Coruzzi et al., 2012).

A growing number of studies have uncovered connections between gut microbiota and the nervous system (Dinan and Cryan, 2017, Sharon et al., 2016). Our exploration of microbiota-encoded neurotransmitter receptor agonists expands the mechanistic evidence for simple biogenic amines serving as potentially widespread modulators of the gut-brain axis (Luqman et al., 2018). These data imply that microbiota-dependent dopaminergic, serotonergic, and histaminergic responses likely represent general signaling events in the GI tract with varying activation profiles, depending on the specific collection of bacteria present in an individual’s microbiome.

### Structurally Distinct Lipids Agonize Diverse GPCRs

**Figure 5:**
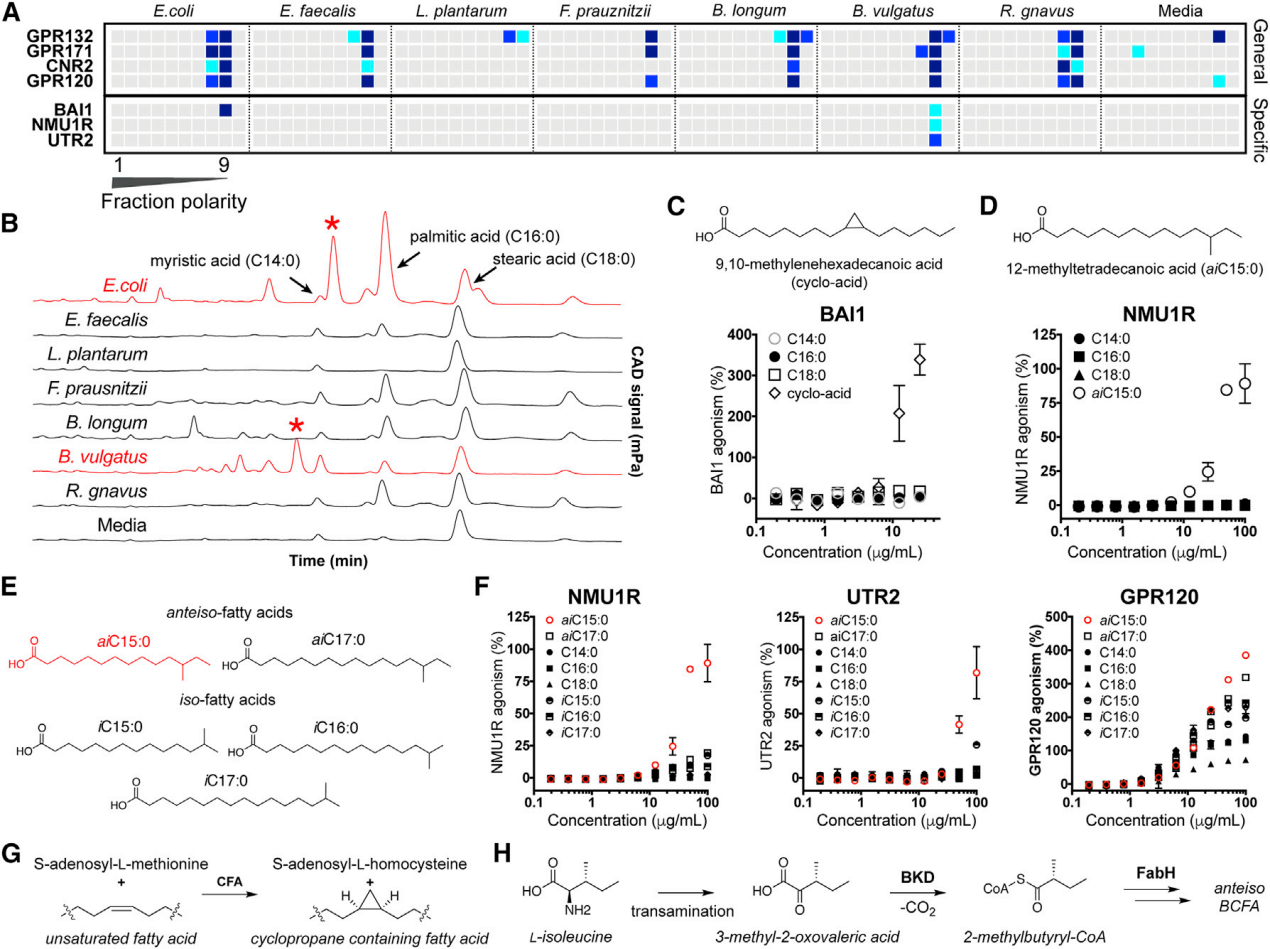
Lipid Responsive GPCRs (A) Heatmap of GPCRs demonstrating general (top) or specific (bottom) responses to lipid-rich fractions of bacterial extracts. (B) Overlaid CAD chromatograms with common lipids and unique lipids (red asterisk) are marked. (C) Structure of BAI1-active lipid 9,10-methylenehexadecanoic acid isolated from *E*. *coli* LF82, and the response of BAI1 to various fatty acids. (D) Structure of NMU1R-active lipid, 12-methyltetradecanoic acid isolated from *B*. *vulgatus*, and the response of NMU1R GPCR to various fatty acids. (E) Panel of branched chain fatty acids tested for GPCR fidelity. (F) Response of NMU1R, UTR2 (specific), and GPR120 (general) to branched chain fatty acid panel. (G) Biosynthesis of cyclopropane rings from unsaturated fatty acids using cyclopropane fatty acid synthase (CFA). (H) Early steps in the biosynthetic scheme for ante-iso branched chain fatty acids (BCFAs) in bacteria (BKD, branched-chain α-keto acid dehydrogenase and FabH, β-ketoacyl-acyl carrier protein synthase III). All dose-response curves were run in duplicate. Error bars are standard deviation. Error bars that are shorter than the height of the symbol are not shown.

Lipids, which represent diverse GPCR-active ligands (An et al., 2014, Round et al., 2011), predominantly elute very late in our fractionation protocol (Figure 5A). Based on the receptor interaction map, we could initially classify GPCRs as lipid responsive if they were agonized by the late lipid-enriched fractions of the extract library. A subset of receptors, including GPR120, CNR2, GPR171, and GPR132, responded broadly to the lipid fraction from most of the consortium, whereas other responses were specific to particular species (BAI1, NMU1R, and UTR2). HPLC-charged aerosol detection analysis of the lipid fractions indicated they contained not only mixtures of simple, saturated fatty acids but also other more complex lipid species (Figure 5B). Marrying unique receptor activity profiles with unique lipid signals guided us to previously unrecognized bacteria-encoded GPCR agonists.

The brain angiogenesis factor 1 (BAI1) receptor was agonized by lipid fractions from the Gram-negative bacteria in the consortium: *E*. *coli* and *B*. *vulgatus*. The *E*. *coli* LF82 lipid fraction showed the most potent agonism of BAI1, and therefore it was selected for further analysis. Bioassay-guided fractionation identified the BAI1 agonist as the cyclopropyl-containing lipid 9,10-methylenehexadecanoic acid (EC_50_, 11 μM). Synthetic 9,10-methylenehexadecanoic acid, but no saturated lipids we tested, agonized BAI1, confirming the specificity of the receptor reflected in the initial GPCR activity map (Figure 5C). The enzyme cyclopropane-fatty-acyl-phospholipid synthase (Cfa) uses the one-carbon donor S-adenosyl-L-methionine to generate cyclopropyl lipids from unsaturated fatty acids (Figure 5G). Cyclopropane-containing fatty acids are important membrane components in Gram-negative as well as mycolic acid bacteria (Table S5) (Wessjohann et al., 2003). Macrophages use BAI1 as a pattern recognition receptor to sense Gram-negative bacteria and to induce selective phagocytosis and antimicrobial responses; 9,10-methylenehexadecanoic acid may represent a previously unrecognized recognition motif for innate immune responses (Billings et al., 2016, Das et al., 2011, Das et al., 2014; ref14; Lee et al., 2016).

Two peptide receptors, neuromedin receptor 1 (NMU1R), which mediates satiety and peristalsis in the gut (Brighton et al., 2004, Howard et al., 2000), and the vasoconstriction inducing urotensin 2 receptor (UTR2), responded specifically to lipid fractions generated from *B*. *vulgatus*. Isolation of the active metabolite yielded the *anteiso*-methyl branched-chain fatty acid 12-methyltetradecanoic acid (aiC15:0) (Figure 5D). *Anteiso*-fatty acids (ai) contain an alkyl branch at the ante-penultimate carbon in contrast to iso-fatty acids (i), which branch at the penultimate carbon. Both synthetic and natural aiC15:0, but no simple fatty acids we tested, agonized NMU1R (EC_50_, 125 μM) and UTR2 (EC_50_, 191 μM). Lipid sensitivity of NMU1R and UTR2 appears specific to aiC15:0, as fatty acids with even slightly modified branching patterns (iC15:0) or carbon chain length (aiC17:0) displayed minimal agonist activity (Figures 5E and 5F). Methyl-branched fatty acids arise from the use of a branched primer in place of acetyl CoA in normal fatty acid biosynthesis. In the case of *anteiso*-methyl-branched fatty acid, 2-methyl-butyryl-CoA, which is derived from isoleucine is used to prime fatty acid biosynthesis (Figure 5G). The selectivity for branched primers lies with the β-ketoacyl acyl carrier protein synthase (KAS III or FABH) that carries out the first condensation in fatty acid biosynthesis. *Anteiso*-methyl fatty acids are predominantly produced by Gram-positive FABH enzymes.(Kaneda, 1991, Lu et al., 2004) Roughly 10% of bacteria have lipid pools enriched in branched chain fatty acids (BCFAs) (Kaneda, 1991). *B*. *vulgatus* is among those bacteria enriched in BCFAs and maintains aiC15:0 as ∼30% of its total fatty acid repertoire (Table S5) (Mayberry et al., 1982).

Bacteria are known to produce diverse and oftentimes taxa-specific collections of lipids. The examples described here from examining even this minimized model microbiome suggest the potential for markedly different receptor activation profiles, and hence biological consequences depending on the specific lipid signature encoded by an individual’s microbiome. For BAI1, NMU1R, and UTR2 our data suggest that they differentially respond to lipids produced by largely Gram-positive or Gram-negative bacteria, indicating that their activities will fluctuate with changes in the gross taxonomic composition of a microbiome.

### Analysis of Mice Colonized with the Seven Strain SIHUMI Consortium

**Figure 6:**
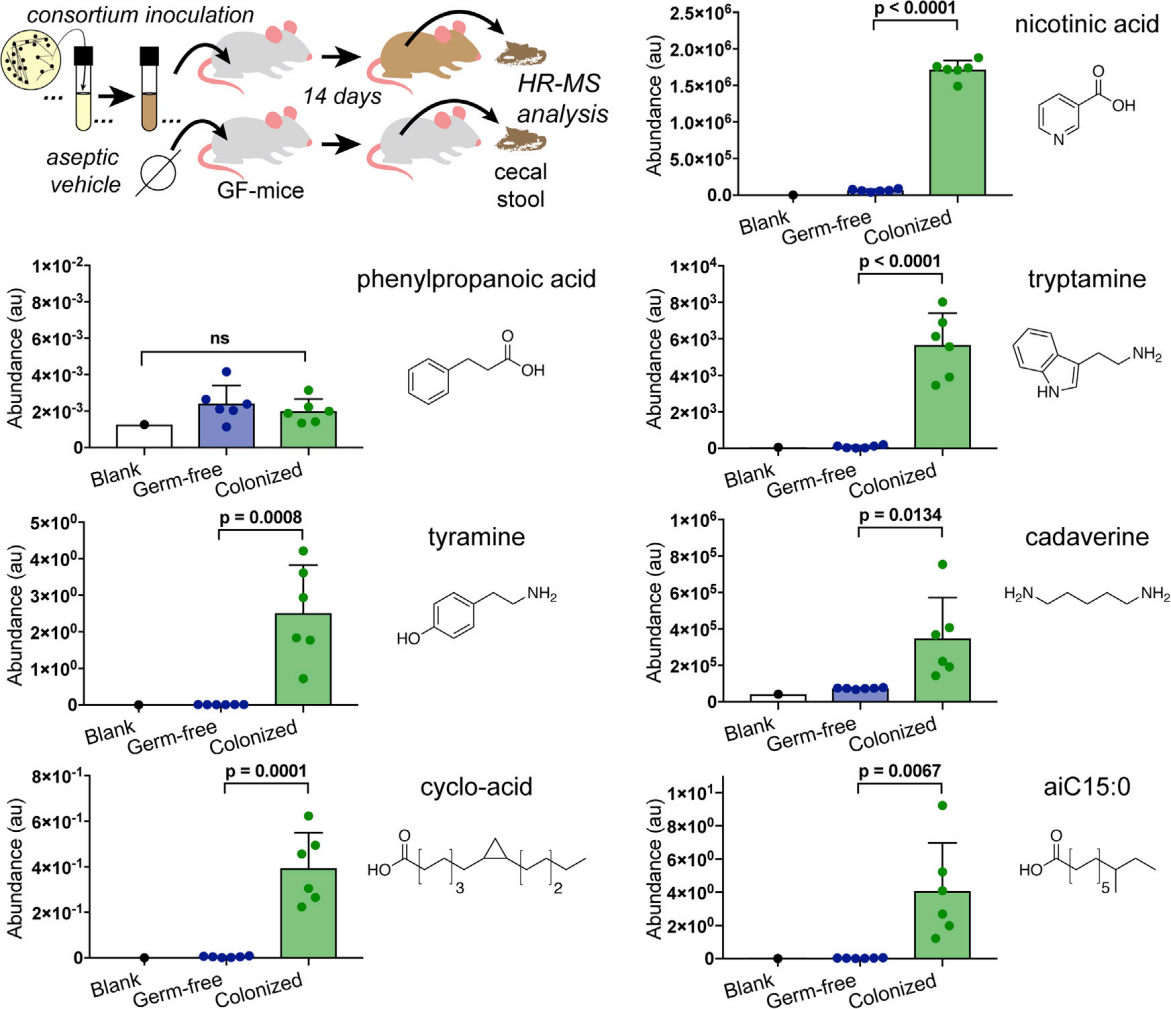
Comparative Analysis of Metabolite Levels in the Cecum of Abiotic Mice to Levels in Mice Inoculated with SIHUMI Consortium Metabolite presence in lumen cecal samples was determined by targeted mass spectrometry. Samples were normalized to each other based on the addition of isotopically labeled internal standards during extraction, n = 6, error bars are standard deviation, p values are derived from the unpaired t test.

In parallel with our *in vitro* screen studies, we used targeted mass spectrometry-based metabolomics to compare germ-free and SIHUMI-consortium-colonized mice. For this analysis, cultures of individually grown bacteria from the consortium were combined and the mixed sample was gavaged into germ-free C57BL/6 mice. PCR-based species analysis of DNA extracted from the stool of animals 3 days post-inoculation confirmed their colonization by the consortium (Figure S5). Ten days post-colonization the lumen material (cecal stool) was collected from the germ-free controls as well as the SIHUMI-colonized animals. Using targeted mass spectrometry, we looked for differences in metabolite accumulation in these samples (Figure 6).

Targeted MS analysis of cecum extracts revealed that all but one of the GPCR-active metabolites we identified was enriched in these mice compared to their abiotic counterparts (Figure 6; Table S6), suggesting a largely parallel biosynthesis in laboratory grown monocultures and the consortium *in vivo*. The lone exception was phenylpropanoic acid. Our inability to detect phenylpropanoic in stool is likely due to low production of this metabolite by the specific strain used in this consortium. This is supported by the low titers we observed *in vitro* (< 0.5 mg phenylpropanoic acid/L) and the low GPR109B activity we observed in our initial fraction screen. However, the low titers observed here do not preclude this metabolite’s potential biologic relevance, especially in light of the fact that it has been shown to be produced by species of the major gut taxa *Firmicutes* and *Bacteroidetes* and has been quantified at μM levels in human stool samples (Gao et al., 2009, Jellet et al., 1980, Rowland et al., 2018, Russell et al., 2013).

## Discussion

Phenylpropanoic acid, cadaverine, 9–10-methylenehexadecanoic acid, and 12-methyltetradecanoic acid add to a growing list of structurally simple molecules that are capable of modulating human signaling pathways that underlie diverse clinically relevant areas of physiology, including immune recognition, neurotransmission, and inflammation (Rooks and Garrett, 2016, Sharon et al., 2016). The biosynthetic simplicity of these metabolites combined with their abundant starting materials and demonstrated roles in fundamental bacterial processes likely drives their high titers in the gut and potential broad biological relevance. Expanding functional screening to include not only more bacteria but also additional culture conditions and receptor families will undoubtedly provide additional insight into the biochemical mechanisms and small molecules underlying human-microbiome interactions. For example, a study that was published while the work reported here was under review examined a different collection of bacteria and identified different GPCR-active metabolites (Chen et al., 2019). Advancements in laboratory culturing techniques now allow for a majority of gut bacteria to be cultured from fecal samples (Browne et al., 2016, Forster et al., 2019). Systematic functional screening of metabolites produced by this growing collection of bacteria is likely to be a rewarding avenue for developing mechanistic hypotheses that can be tested in specific animal models.

## STAR★Methods

### Key Resources Table

**Table:** 

REAGENT or RESOURCE	SOURCE	IDENTIFIER
**Bacterial and Virus Strains**		

Escherichia coli LF82	Gift of Daniel Mucida	N/A
Enterococcus faecalis OG1RF	Gift of Daniel Mucida	N/A
Lactobacillus plantarum WCFS1	Gift of Daniel Mucida	N/A
Faecalibacterium prausnitzii A2-165	Gift of Daniel Mucida	N/A
Bifidobacterium longum ATCC 15707	Gift of Daniel Mucida	N/A
Bacteroides vulgatus ATCC 8482	Gift of Daniel Mucida	N/A
Ruminococcus gnavus ATCC 29149	Gift of Daniel Mucida	N/A

**Chemicals, Peptides, and Recombinant Proteins**		

tyramine	Alfa Aesar	CAT#: A12220
tryptamine	Alfa Aesar	CAT#: A11030
12-methyltetradecanoic acid	ChemCruz	CAT#: sc-213601
cis-9,10-methylenehexadecanoic acid	Avanti Polar Lipids, Inc.	CAT#: 857500C
cadaverine	Sigma Aldrich	CAT#: C8561
nicotinic acic	Sigma Aldrich	CAT#: N4126
hydrocinnamic acid	Sigma Aldrich	CAT#: 135232
phenol-D6	Cambridge Isotope Laboratories	CAT#: DLM-370-5
palmitic acid-d31	Cambridge Isotope Laboratories	CAT#: DLM-215

**Critical Commercial Assays**		

ZymoBIOMICS DNA/RNA Miniprep Kit	ZYMO Research	CAT#: R2002
PathHunter screen	Eurofins DiscoverX	N/A

**Deposited Data**		

Protein expression data	Human Protein Atlas (Uhlén et al., 2015)	https://www.proteinatlas.org
Human Microbiome Project Genome Database	Human Microbiome Project Consortium, 2012	https://hmpdacc.org

**Experimental Models: Organisms/Strains**		

Germ free C57BL/6 mice	Gnotobiotic facility and Microbiome Translational Center	N/A

**Oligonucleotides**		

27F: AGAGTTTGATCMTGGCTCAG	Integrated DNA Technologies	N/A
1492R: GGTTACCTTGTTACGACTT	Integrated DNA Technologies	N/A
Escherichia coli LF82 FWD: GTTAATACCTTTGCTCATTGA	Integrated DNA Technologies	N/A
Escherichia coli LF82 REV: ACCAGGGTATATAATCCTGTT	Integrated DNA Technologies	N/A
Enterococcus faecalis OG1RF FWD: CCCTTATTGTTAGTTGCCATCATT	Integrated DNA Technologies	N/A
Enterococcus faecalis OG1RF REV: ACTCGTTGTACTTCCCATTGT	Integrated DNA Technologies	N/A
Lactobacillus plantarum WCFS1 FWD: AGCAGTAGGGAATCTTCCA	Integrated DNA Technologies	N/A
Lactobacillus plantarum WCFS1 REV: CACCGCTACACATGGAG	Integrated DNA Technologies	N/A
Faecalibacterium prausnitzii A2-165 FWD: CCCTTCAGTGCCGCAGT	Integrated DNA Technologies	N/A
Faecalibacterium prausnitzii A2-165 REV: GTCGCAGGATGTCAAGAC	Integrated DNA Technologies	N/A
Bifidobacterium longum ATCC 15707 FWD: GGGTGGTAATGCCGGATG	Integrated DNA Technologies	N/A
Bifidobacterium longum ATCC 15707 REV: TAAGCGATGGACTTTCACACC	Integrated DNA Technologies	N/A
Bacteroides vulgatus ATCC 8482 FWD: GGTGTCGGCTTAAGTGCCAT	Integrated DNA Technologies	N/A
Bacteroides vulgatus ATCC 8482 REV: CGGAYGTAAGGGCCGTGC	Integrated DNA Technologies	N/A
Ruminococcus gnavus ATCC 29149 FWD: CGGTACCTGACTAAGAAGC	Integrated DNA Technologies	N/A
Ruminococcus gnavus ATCC 29149 REV: AGTTTYATTCTTGCGAACG	Integrated DNA Technologies	N/A

**Software and Algorithms**		

Prism 7	Graphpad	https://www.graphpad.com/scientific-software/prism/
XCalibur	Thermo Scientific	https://www.thermofisher.com/order/catalog/product/OPTON-30487
MassLynx	Waters Instruments	https://www.waters.com/waters/en_US/MassLynx-Mass-Spectrometry-Software-/nav.htm?cid=513164&locale=en_US
MassHunter	Agilent Technologies	https://www.agilent.com/en/products/software-informatics/masshunter-suite/masshunter/masshunter-software
TraceFinder	Thermo Scientific	https://www.thermofisher.com/order/catalog/product/OPTON-30491
MestReNova	Mestrelab Research	https://www.mestrelab.com

### Lead Contact and Materials Availability

Further information and requests for reagents may be directed to and will be fulfilled by the Lead Contact, Sean F. Brady (sbrady@rockefeller.edu).

#### Materials Availability Statement

This study did not generate new unique reagents.

### Experimental Model and Subject Details

#### Mouse Studies

In-house bred germ free C57BL/6 mice were maintained in sterile isolators with autoclaved food and water in the Gnotobiotic Facility of the Faith Lab at Mount Sinai. 6-week-old mice were used for all experiments (3M and 3F in the treatment group, 5M and 1F in the control group). All experimental procedures were approved by the Animal Care and Use Committee of The Icahn School of Medicine at Mount Sinai (PI Cohen IACUC-2016-0491).

#### Microbe Strains

Details of SIHUMI microbe strains can be found in the Key Resources Table. Anaerobic bacteria were cultured in an incubator set to 37°C placed inside of vinyl anaerobic chamber (Coy) with a gas mix of 5% CO_2_, 5% H_2_, and 90% N_2_. The cadA-KO strain of E. coli LF82 was grown aerobically at 37°C in LB with indicated antibiotics during genetic manipulation for practicality.

### Method Details

#### Media Construction

LBM media: LBM media was derivative of a media recipe previously utilized in our laboratory. For 1 L media: Bring 17 g/L brain heart infusion, 5 g/L yeast extract, 200 mg MgSO_4_⋅7H_2_O, 100 mg MnCl_2_·4H_2_O up in 800 mL deionized water and autoclave for 30 minutes liquid cycle. After coming to room temp, add supplements (final concentrations: 5 μg/L hemin, 1 g/L maltose, 1 g/L cellobiose, and 0.5g/L L-cysteine), which can be made ahead of time and stored, protected from light, at -20°C in aliquots, except hemin which can be stored at 4°C and L-cysteine which should be made fresh. Use autoclaved deionized water to bring final volume to 1 L. For culturing anaerobes: place media in anaerobic chambers for at least 48 hrs to allow diffusion with anaerobic gas. When assessing cadaverine induction, media was acidified with HCl then sterile filtered.

#### Cultivation of Bacteria

Bacterial strains of the SIHUMI consortium used listed in the Key Resources Table. Cultures of <1 L: anaerobic bacteria were cultured in an incubator set to 37°C placed inside of vinyl anaerobic chamber (Coy) with a gas mix of 5% CO_2_, 5% H_2_, and 90% N_2_. Cultures of >1 L: When cultivating bacteria for construction of bacterial extract library, bacteria were inoculated in 1 L or 2 L media bottles (Chemglass) inside anaerobic chamber, then sealed with anaerobic septa (Chemglass) and moved into large walk-in 37°C incubator constructed from a 6x6x12 light protective tent (APOLLO® HORTICULTURE) outfitted with a regulator (INKBIRD®), heat source (VORNADO®), and ventilation system (IPOWER®). Freezer stocks of the SIHUMI cohort were generously donated by the Mucida laboratory (Rockefeller University). Freezer stocks were thawed and bacteria were cultivated overnight in LBM media until turbid. These bacteria were streaked onto LBM agar plates and upon growth, single colonies were picked, cultivated overnight, and genotyped (GeneWiz). Upon confirmation of genetic identity, these same cultures were used to generate colony 20% glycerol stocks that would be used for the entirety of the study. Strain specific primers were used to allow PCR-based identification of each strain and are listed in the Key Resources Table. For large scale fermentations the following protocol was used: Bacterial stocks were thawed and used to inoculate 5 mL LBM liquid cultures that were cultivated overnight. The next day, species specific primers were used to confirm identity (as described below) and upon passing purity check, these 5 mL cultures were used to inoculate 500 mL LBM at a ∼1:100 ratio. After turbidity was reached, an aliquot of the 500 mL culture was removed and PCR was performed with universal 16s rRNA primers 27F and 1492R (sequences in Key Resources Table). The PCR product was subject to Sanger sequencing (GeneWiz) and upon passing inspection for the correct species, the 500 mL culture was used to inoculate 12 L of LBM media at a 1:100 inoculation ratio. The 20 L cultures were cultivated, protected from light at 37°C, for 10 days without shaking. Amerlite XAD-7HP (Sigma Aldrich) was aliquoted in 20 g increments and activated by soaking in methanol for 10 minutes, followed by 5 washes with deionized water to remove excess methanol. After 10 days, activated Amberlite XAD-7HP was added to the cultures (20 g dry weight/L) and the slurries were gently shaken (90 rpm) on a tabletop shaker for 4 hrs. After incubation with the cultures, the resin was removed via cheese-cloth filtration and the collected resin. alongside the cheese-cloth, was placed inside a 1 L Fernbach flask to which 1.5 L acetone was added. This acetone elution was allowed to occur for 2 hrs with shaking (150 rpm), after which the organic solvent was collected and fresh acetone, of equivalent volume, was added. This second elution was allowed to occur overnight with light shaking at 22°C. Both elutions were added together and solvent was removed via rotary evaporation (Buchi) at 25°C to afford the dry crude extract, which was stored at -20°C until fractionated, as detailed below.

#### Fractionation of Bacterial Extracts

Crude extracts (∼1-3 g/12L) were re-suspended in ∼300 mL methanol and the soluble material was decanted into a 500 mL round bottom flask (rbf). Free C_18_ resin (2-3 g) was added and the slurry was evaporated under reduced pressure using a rotary evaporator with temperature set to 25°C (Buchi). The dry material was collected from the rbf, packed semi-tightly into a 50 g cartridge, and capped with a passive frit (Teledyne). This material was chromatographed over a 150 g C18 Gold (Teledyne ISCO) using a solvent system of water (Solvent A) and methanol (Solvent B), with no acid added, with the following conditions. 5 column volumes (CV) of 5% B, 5% B to 99% B over 10 CV, flush with 10 CV 99% B. All flow-through was collected in 50 mL tubes and combined as follows:

Solvent was evaporated using an SPD-2010 speedvac (Thermo Scientific) with a RH-19 rotor (Thermo Scientific) and the resulting dry material was weighed and resuspended at 100 mg/mL using ACS grade DMSO (Fisher Scientific). Of this solution, 250 μL was removed and added to 250 μL DMSO to create 500 μL 50 mg/mL solution; this solution was aliquoted into various sizes of 96-well plates for facile thawing and biological testing at a later time. The remaining 100 mg/mL solution was stored at -80°C until validation studies required material for bio-assay guided fractionation.

#### GPCR Assays

GPCR activities were measured by Eurofins DiscoverX using the PathHunter® β-Arrestin assay (Olson and Eglen, 2007). This assay uses β-galactosidase (β-Gal) that has been split into two inactive portions as a reporter to measure the activation of a GPCR. The β-Gal fragments are called EA for Enzyme Acceptor and ED for Enzyme Donor.(US Patent: US20090098588A1) Using these fragments, a unique reported cell line was created for each GPCR of interest. In each unique cell line the EA fragment is fused to β-Arrestin and the ED fragment is fused to the GPCR of interest. Upon GPCR activation, β-Arrestin recruitment to the receptor physically colocalizes the ED and EA fragments thereby restoring β-Gal activity. β-Gal complementation is measured using chemiluminescent PathHunter® Detection Reagents. For our initial screen (Figure 2) all 80 culture broth extract fractions were screened in singleton against the Eurofins DiscoverX gpcrMAX (**168 GPCRs**, Table S2) and orphanMAX (73 GPCRs, Table S2) panels. All subsequent validation and bioassay guided fraction studies were run in at least duplicate using individual reporter cell lines for specific GPCRs of interest.

##### Eurofins DiscoverX Generic Agonist Protocol

1. Sample is added to individual GPCR reporter cell lines grown in microtiter plates. Cells are incubated at 37°C or room temperature for 90 or 180 minutes. 2. Assay signal is generated through addition of 12.5 or 15 μL (50% v/v) of PathHunter Detection reagent cocktail, followed by incubation for a one hour hours at room temperature. 3. Microplates are read with a PerkinElmer EnvisionTM instrument for chemiluminescent signal detection. 4. Compound activity is analyzed using the CBIS data analysis suite (ChemInnovation, CA). For receptors with known ligands, percentage activity is calculated using the following formula: % Activity =100% x (mean RLU of test sample — mean RLU of vehicle control) / (mean MAX control ligand — mean RLU of vehicle control). For orphan receptors, percentage inhibition is calculated using the following simplified formula: % Activity =100% x (mean RLU of test sample — mean RLU of vehicle control) / (mean RLU of vehicle control). For some orphan receptors that exhibit low basal signal, the more sensitive PathHunter Flash Kit is used.

#### Biosynthesis Analysis

Using the annotated genome of *E*. *coli* LF-82 the Pfam protein features of CadA (Accession ID LF82_0254) or the amino acid sequence itself were used as a query against the annotated genome collection provided by the NIH Human Microbiome Project (Human Microbiome Project Consortium, 2012, The Human Microbiome Project et al., 2012). This dataset was chosen as it allowed us to confidently assign annotated bacterial genomes containing a *cadA* gene to body sites origin. For the Pfam analysis: three Pfam motifs are found within the CadA amino acid sequence: PF01276 constitutes the major domain of the decarboxylase, PF03711 constitutes the C-terminal domain and PF03709 constitutes the N-terminal domain. The raw data for both BlastP (>30% identity) and Pfam-based analyses are available in Table S4. The E. coli LF82 gene *cfa* and B. vulgatus E1 subunit of the BKD complex (alpha-ketoacid dehydrogenase subunit alpha/beta) were used in similar BlastP analyses.

#### Mutation of *cadA* in *E*. *coli* LF82

Deletion of the *cadA* gene was performed by Red/ET recombination. *E*. *coli* LF82 cells were transformed with the pRedET plasmid (Genebridges) and grown overnight at 30°C on LB agar plates supplemented with 3 μg/mL tetracycline. A single colony was picked and grown overnight at 30°C in 5 mL LB, followed by 100-fold dilution in 50 mL fresh LB. This culture was grown at 30°C to OD600 0.3, at which point L-arabinose was added to a final concentration of 0.4 wt% to induce recombination-mediating proteins. The culture was grown for 1 hr at 37°C before making the cells electrocompetent. These cells were transformed with 200 ng of a linear piece of DNA bearing an apramycin resistance cassette flanked by 250 bp regions upstream and downstream of the *cadA* gene. Recombination was allowed to occur for 3 hr at 37°C before plating the cells on LB agar supplemented with 50 ug/mL apramycin and growing overnight at 37°C. Colony PCR was used to check for the appropriate gene deletion and apramycin cassette insertion.

#### Cadaverine Induction

LB was acidified to indicated pH using 1N HCl then sterile filtered. In duplicate, 5 mL of media was inoculated by pipetting 50 uL of turbid liquid cultures of either wild-type *E*. *coli* LF82 or cadA-KO *E*. *coli* LF82. Cultures were allowed to grow overnight at 37°C. Turbid cultures were centrifuged at 4,500 rpm for 10 minutes to pellet cells, the dry weight of which was used for normalization between samples. The supernatant was moved to a 16 mL glass tube and the pH was raised to ∼11 with 1N sodium hydroxide. Basified supernatants were then extracted 1:1 with ethyl acetate one time and the organic layer was dried under nitrogen gas. To each vial was added 100 uL of 50% acetonitrile in water and samples were run and analyzed using HR-MS techniques described below. A synthetic standard of cadaverine was used to set analysis parameters including retention time and accurate mass.

#### High-Resolution Mass Spectrometry

High Resolution Mass Spectrometry was used in structure elucidation of pure, unknown compounds as well as relative quantification between samples. HRMS was acquired on a C18 column (Thermo Acclaim 120 C_18_, 2.1 × 150 mM) using a Dionex U-3000 HPLC system connected to an LTQ-Orbitrap Mass Spectrometer (Thermo-Fisher). Analysis was performed using Thermo Xcalibur.

#### Murine Work

All experimental procedures were approved by the Animal Care and Use Committee of The Icahn School of Medicine at Mount Sinai (PI Cohen IACUC-2016-0491). Germ free C57BL/6 mice were maintained in sterile isolators with autoclaved food and water in the Gnotobiotic Facility of the Faith Lab at Mount Sinai. 6-week-old mice were used for all experiments (3M and 3F in the treatment group, 5M and 1F in the control group). The treatment group was colonized with the SIHUMI whereas the control group was left germ free. For colonization studies 5 ml of an overnight culture in LBM media of the SIHUMI (treatment group) was centrifuged at 500 x g for 2 minutes, the supernatant was decanted and the cells were resuspended in 2 ml of sterile PBS. Germ free mice were gavaged with 100 μL of bacterial culture immediately upon removal from sterile isolators. Colonization was confirmed by collection of fecal pellets after 3 and 10 days. Crude DNA was extracted from fecal pellets per protocol (ZymoBIOMICS DNA/RNA Miniprep Kit) and colonization conformed by targeted PCR of each strain using specific primers as detailed above (Figure S5). After colonization mice were housed in specific-pathogen-free conditions and fed with autoclaved water and food. After colonization for 10 days the mice were euthanized and samples were collected for analysis. 200 mg of cecal contents were collected from each mouse and placed immediately at -80°C. The animal experiments were not randomized and the investigators were not blinded to the allocation during experiments and outcome assessment. No statistical methods were used to predetermine sample size. All mice which completed the experiments were analyzed.

#### Metabolite Quantitation by Mass Spectrometry

Cecal samples were weighed into 2 mL microtubes containing 2.8 mm ceramic beads (Omni International) and resuspended to a final concentration of 100 mg/mL using 80:20 methanol:water containing phenol-d6, palmitic acid-d31 and ^13^C,^15^N-amino acid internal standards (Cambridge Isotope Laboratories). Homogenization was using a Bead Ruptor (Omni International) at 6 m/s for 30 s for 6 cycles, at 4°C. Samples were centrifuged for 20 minutes at 20,000 x g at 4°C and then divided for 3 analytical methods.

Estimated concentrations of all metabolites (Table S6) were determined by preparing a calibration curve of pure standards in 80% MeOH and processed as described below for Methods 1-3. Calibration curves were 1/x^2^-weighted and absolute ranges (in μM) were defined where the accuracy of the calibrators were calculated to be within +/- 30% of the nominal value.

#### Method 1: GC-nCI-MS with PFB Derivatization

100 μL of cecal extract was added to 100 μL of 100 mM borate Buffer (pH 10), 400 μL of 100 mM pentafluorobenzyl bromide (Thermo Scientific) in acetone (Fisher), and 400 μL of cyclohexane (Acros Organics) in a sealed autosampler vial. Samples were heated to 65°C for 1 hour with shaking. After cooling to room temperature and allowing the layers to separate, 100 μL of the cyclohexane upper phase was transferred to an autosampler vial containing a glass insert and sealed. Samples were analyzed using a GC-MS (Agilent 7890A GC system, Agilent 5975C MS detector) operating in negative chemical ionization mode, using a DB-5MS column (30 m x 0.25 mm, 0.25, 0.25 μm; Agilent Technologies), methane as the reagent gas and 1 μL split injection (1:5 split ratio). Raw peak areas for aromatic analytes (tyramine and phenylpropanoic acid) were normalized to phenol-d7 internal standard and lipid analytes (9,10-methylenehexadecanoic acid and 12-methyltetradecanoic acid) were normalized to palmitic acid-d31 internal standard. Data analysis was performed using MassHunter Quantitative Analysis software (version B.09, Agilent Technologies) and confirmed by comparison with authentic standards.

#### Method 2: LC triple quadrupole with reverse phase chromatography

200 μL of extract was dried using a vacuum concentrator (Genevac) and resuspended in 200 μL 50:50 methanol:water, clarified by centrifugation and analyzed using reverse phase chromatography coupled to TSQ Vantage triple quadupole mass spectrometer with HESI II source. LC separation was using an HSS T3 column (100 x 2.1 mm, 1.8 μm particle size, Waters) and Agilent 1260 binary pump. Mobile phase A was 0.1% formic acid in water and mobile phase B was 0.1% formic acid in acetonitrile. The gradient was 0 min, 0% B; 2 min, 0% B; 5 min, 12% B; 7  min, 70% B; 8.5  min, 97% B, 11.5 min, 97% B with 3.5 min of re-equilibration time. LC parameters were: flow rate 300 μL/min, injection volume 15 μL and column temperature 35°C. The mass spectrometer was operated in positive ionization with transitions for tyrptamine (m/z 161.1 → 115.1, CE 30V^∗^; 161.1 → 144.1, CE 4 V) and nicotinic acid (m/z 124.1 → 80.1, CE 18 V^∗^; 124.1 → 78.1, CE 19V), with ^∗^ indicating the primary transition used for quantitation. MS parameters were: capillary temp: 300°C; vaporizer temp: 350°C; sheath gas: 50; aux gas: 30; spray voltage 4000 V. Data was acquired and analyzed using TraceFinder software (version 4.1, Thermo Scientific) confirmed by comparison with authentic standards.

#### Method 3: LC Q-TOF with HILIC chromatography

Samples were prepared as for Method 2, with 100 μL of the 50:50 methanol:water extract added to an additional 100 μL 60:40 acetonitrile:water and analyzed by hydrophilic interaction chromatography (HILIC) coupled to the 6545 Q-TOF mass spectrometer with Dual JetStream source (Agilent). The LC separation was using an Acquity UPLC BEH Amide column (150 mm × 2.1 mm, 1.7 μm particle size, Waters) and Agilent 1290 Infinity II binary pump. Mobile phase A was 90:10 water:acetonitrile with 10 mM ammonium acetate and 0.2% acetic acid, and mobile phase B was 10:90 water:acetonitrile with 10 mM ammonium acetate and 0.2% acetic acid. The gradient was 0 min, 95% B; 9 min, 70% B; 10 min, 40% B; 13 min, 30% B; 15 min, 95% B. LC parameters were: flow rate 400 μL/min, column temperature 40°C, and injection volume 5 μL. The mass spectrometer was operated in positive ionization mode. MS parameters were: gas temp: 325°C; gas flow: 10 L/min; nebulizer pressure: 35 psig; sheath gas temp: 400°C; sheath gas flow: 12 L/min; VCap: 4,000 V; fragmentor: 125 V. Active reference mass correction was done through a second nebulizer using masses with m/z: 121.050873 and 922.009798. Data were acquired over m/z range 50–1700 and analyzed using MassHunter Profinder software (version B.09, Agilent) and confirmed by comparison with a cadaverine authentic standard. Compiling these data sets in GraphPad Prizm was then used to derive p-values. Unpaired t-test (two-tailed) were used.

#### Tyramine

Fraction 3 from *E*. *coli* LF82 was chosen as the pilot fraction for the dopamine receptors. 1 mL of Fraction 3 (100 mg/mL in DMSO) was dried down resuspended in 1 mL 50/50 MeOH:H2O and injected in 50 μL increments onto a semi-preparative 250 x 10 mm Luna® Omega 2.6 uM Polar C18 LC column on an Agilent 1100 HPLC with a solvent system where Solvent A was H_2_O + 0.1% formic acid and Solvent B was CH_3_CN + 0.1% formic acid. The chromatographic method was as follows: 0% B for 5 CV, then up to 90% B over 15 CV, with a 5 CV hold at 90% B. Peak detection and fraction collection was driven by UV absorbance at 210 nm, 254 nm, 280 nm, and 330 nm. Fractions were collected and re-assayed against DRD3 to guide further purification. The active fraction was further purified using a 150 x 10 mm Kinetix® 5 μm Biphenyl 100A LC column. A single resulting fraction retained activity and this compounds was identified as tyramine by NMR and HRMS (LC-HRMS-ESI (*m/z*): [M+H]^+^ calcd for C_8_H_11_NO, 138.0841; found 138.0911). Tyramine: ^1^H NMR (DMSO-*d*_6_, 600 MHz): δ_H_ 7.02 (2H, d, *J* = 8.5 Hz), 6.70 (2H, d, J = 8.5 Hz), 2.89(2, t, J = 7.6 Hz), 2.71 (2H, t, J = 7.6 Hz). ^13^C NMR (DMSO-*d*_6_, 151 MHz): δ_C_ 156.1 (1C, s), 129.5 (2C, s), 115.3 (2C, s), 40.8 (1C, s), 33.45 (1C, s).

#### Tryptamine

A single fraction from the fermentation of *Ruminococcus gnavus* was chosen as a pilot fraction to find serotonin active compounds which could then be assessed in other bacteria. 1 mL of Fraction 5 solution (100 mg/mL in DMSO) was dried down, resuspended in 1 mL 50/50 MeOH:H_2_O, and injected in 50 μL increments onto a semi-preparative 250X10 mm Luna® Omega 2.6 μM Polar C18 LC column on an Agilent 1100 HPLC with a solvent system where Solvent A was H2O + 0.1% formic acid and Solvent B was CH3CN + 0.1% formic acid. The chromatographic method was as follows: 0% B for 5 CV, then up to 90% B over 15 CV, with a 5 CV hold at 90% B. Peak detection and fraction collection was driven by UV absorbance at 210 nm, 254 nm, 280 nm, and 330 nm. Fractions were collected and re-assayed against HTR5A to guide further purification. The active fraction (41 mg) was ∼90% tryptamine as evident by NMR and HRMS (LC-HRMS-ESI (*m/z*): [M+H]^+^ calcd for C_8_H_11_NO, 161.1000; found 161.1071). Tryptamine: ^1^H NMR (DMSO-*d*_6_, 600 MHz): δ_H_ 7.54 (1H, d, *J* = 7.9 Hz), 7.20 (1H, d, 2.0 Hz), 7.08 (1H, t, 7.6 Hz), 7.00 (1H, t, 7.6 Hz), 3.01 (2H, dd, 8.5 Hz, 7.1 Hz), 2.93 (2H, dd, 8.8, 6.2 Hz). ^13^C NMR (DMSO-*d*_6_, 151 MHz): δ_C_ 136.3 (1C, s), 126.9 (1C, s), 123.2 (1C, s), 121.1 (1C, s), 118.4 (1C, s), 118.1 (1C, s), 111.5 (1C, s), 110.1 (1C, s), 40.0 (1C, s), 24.6 (1C, s).

#### Phenylpropanoic acid

Due to its relative simplicity in composition, Fraction 2 was chosen for further study. 40 mg of Fraction 2 was injected in two equal increments onto a semi-preparative 150 x 10 mm XBridge® 5 μm C18 columnon an Agilent 1100 HPLC with a solvent system where Solvent A was H_2_O + 0.1% formic acid and Solvent B was CH_3_CN + 0.1% formic acid. The chromatographic method was as follows: flow rate 4 mL/min; 2.5% B for 5 min, then increased to 35% B over 25 min, then flushed at 99% B for 5 min. Peak detection and fraction collection was driven by UV absorbance at 210 nm, 254 nm, 280 nm, and 330 nm. Fractions were collected per minute and re-assayed against GPR109B. All activity was found in subfractions 30 (8.8 mg) and 31 (0.1 mg), which were identified as pure phenylpropanoic acid by NMR and MS. Significant quantities of phenylpropanoic acid was also subsequently detected in Fraction 7 from all bacterial extracts. LC-HRMS-ESI (*m/z*): [M-H]^-^ calcd for C_9_H_9_O_2_ 149.0602; found 149.0599.^1^H NMR (DMSO-*d*_6_, 600 MHz): δH 12.17 (1H, bs), 7.27 (2H, t, J = 6.9 Hz), 7.22 (2H, d, J = 7.3 Hz), 7.18 (1H, t, J = 7.0 Hz), 2.81 (2H, t, J = 7.8 Hz), 2.51 (2H, t, J = 7.9 Hz). ^13^C NMR (DMSO-d_6_, 151 MHz): δC 174.0 (1C, s), 141.0 (1C, s), 128.3 (2C, s), 128.2 (2C, s), 125.9 (1C, s), 35.5 (1C, s), 30.5 (1C, s).

#### Cadaverine

Fraction 4 from *E*. *coli* LF82 was chosen as the pilot fraction for the histamine receptors. 1 mL of Fraction 4 (100 mg/mL in DMSO) was dried down resuspended in 1 mL H2O and injected in 50-μL increments onto a semi-preparative 250 x 10 mm Luna® Omega 2.6 uM Polar C18 LC column on an Agilent 1100 HPLC with a solvent system where Solvent A was H2O + 0.1% formic acid and Solvent B was CH3CN + 0.1% formic acid. The chromatographic method was as follows: 0% B for 10 CV, then up to 90% B over 5 CV, with a 3 CV hold at 90% B. Peak detection and fraction collection was driven by charged aerosol detection using a Corona Veo (Thermo Fisher Scientific) after UV proved to not be useful. Fractions were collected and re-assayed against HRH4 to guide further purification. The active fraction was further purified two more times using the same Polar C18 column with extended flushes at 0% B, as the activity always was eluting in the void. A HILIC method proved to be less effective. A single resulting fraction retained activity and this compound was identified as cadaverine by NMR and HRMS (LC-HRMS-ESI (*m/z*): [M+H]^+^ calcd for C_5_H_14_N_2_, 102.1157; found 102.12293). Co-eluted in this fraction was the compound agmatine (LC-HRMS-ESI (*m/z*): [M+H]^+^ calcd for C_5_H_14_N_4_, 130.12184; found 131.12920). Cadaverine: ^1^H NMR (D_2_O, 600 MHz): δ_H_ 3.04 (4H, t, *J* = 7.6 Hz), 1.74 (4H, p, *J* = 7.7 Hz), 1.49 (2H, p, *J* = 7.7 Hz). ^13^C NMR (D_2_O, 151 MHz): δ_C_ 39.2 (2C, s), 26.2 (2C, s), 22.7 (1C, s).

#### 9,10-methylenehexadecanoic acid

Fraction 9 of *E*. *coli* LF-82 was injected in DMSO onto a semi-preparative 150 x 10 mm XBridge® 5 μm C18 column with a solvent system where Solvent A was H2O + 0.1% formic acid and Solvent B was CH3CN + 0.1% formic acid. The chromatographic method was as follows: 30% B for 3 column CV then up to 99% B over 5 CV, with a 15 CV hold at 99% B. Peak detection and fraction collection was driven by charged aerosol detection using a Corona Veo (Thermo Fisher Scientific) after UV proved to not be useful. Fractions were collected and re-assayed against BAI1 to guide further purification. A single resulting fraction retained activity and this compound was identified as 9,10-methylenehexadecanoic acid by NMR and HRMS (LC-HRMS-ESI (*m/z*): [M+H]^+^ calcd for C_17_H_32_O_2_, 267.2402; found 267.2334). 9,10-methylenehexadecanoic acid: ^1^H NMR (CDCl_3_, 600 MHz): δ_H_ 2.35 (2H, t, *J* = 7.4 Hz), 1.64 (2H, p, *J* = 7.4 Hz), 1.37 (16H, m), 1.32 (2H, m), 1.14 (2H, m), 0.89 (3H, t, *J* = 6.6 Hz), 0.65 (2H, m), 0.57 (1H, td, J = 8.2 Hz, 4.2 Hz), -0.33 (1H, q, J = 5.2, 4.4 Hz). ^13^C NMR (CDCl_3_, 151 MHz): δC 177.7 (1C, s), 33.8 (1C, s), 32.2 (1C, s), 30.4 (1C, s), 30.4 (1C, s), 29.7 (1C, s), 29.6 (1C, s), 29.5 (1C, s), 29.3 (1C, s), 29.0 (1C, s), 28.9 (1C, s), 25.0 (1C, s), 23.0 (1C, s), 16.0 (1C, s), 16.0 (1C, s), 14.4 (1C, s), 11.2 (1C, s).

#### 12-methyltetradecanoic acid

Fraction 9 of *B*. *vulgatus* was injected in DMSO onto a semi-preparative 150 x 10 mm XBridge® 5μm C18 column with a solvent system where Solvent A was H2O + 0.1% formic acid and Solvent B was CH3CN + 0.1% formic acid. The chromatographic method was as follows: 30% B for 3 column CV then up to 99% B over 5 CV, with a 15 CV hold at 99% B. Peak detection and fraction collection was driven by charged aerosol detection using a Corona Veo (Thermo Fisher Scientific) after UV proved to not be useful. Fractions were collected and re-assayed against BAI1 to guide further purification. A single resulting fraction retained activity and this compound was identified as 9,10-methylenehexadecanoic acid by NMR and HRMS. (LC-HRMS-ESI (*m/z*): [M-H]^-^ calcd for C_15_H_30_O_2_, 241.2245; found 241.2178) 12-methylmyristic acid: ^1^H NMR (CDCl_3_, 600 MHz): δH 2.35 (2H, t, 7.5 Hz), 1.64 (2H, p, 7.5 Hz), 1.26 (16H, m), 1.12 (1H, m, 6.9 Hz), 1.08 (2H, m), 0.85 (3H, t, 7.4 Hz), 0.84 (3H, d, 5.1 Hz). ^13^C NMR (CDCl3, 151 MHz): δC 178.2 (1C, s), 36.6 (1C, s), 34.3 (1C, s), 33.7 (1C, s), 30.0 (1C, s), 29.6 (1C, s), 29.5 (1C, s), 29.4 (1C, s), 29.4 (1C, s), 29.2 (1C, s), 29.0 (1C, s), 27.0 (1C, s), 24.6 (1C, s), 19.2 (1C, s), 11.4 (1C, s).

### Quantification and Statistical Analysis

Statistical analysis was performed using Prism (Graphpad). Statistical parameters including precision measures (mean ± SD) and statistical significance are reported in the Main text, Figures and Figure Legends. Data was judged to be statistically significant when p < 0.05 by unpaired t test (two-tailed).

### Data and Code Availability

All raw screening data is included in Table S2. Screenshots of raw NMR data for isolated compounds are included in Figures S6–S11.

## Supplemental Information

Document S1. Figures S1–S11

Table S1. List of Control Compounds Used for GPCR Agonist Screen, Related to Figure 1

Table S2. Raw Screening Data, Related to Figure 2

Table S3. List of Hit Filtering by Receptor Type, Related to Figure 2

Table S4. Raw cadA (E. coli LF82) BLAST Analysis of HMRGD and PFAM Analysis of HMRGD, Related to Figure 4

Table S5. Raw Cfa (E. coli LF82) BLAST Analysis of HMRGD and Raw BKD.E1 (*B*. *vulgatus* and *B*. *dorei*) BLAST Analysis of HMRGD, Related to Figure 5

Table S6. Raw Targeted Mass-Spectrometry Metabolomics Data and Estimated Absolute Concentrations for Targeted Mass-Spectrometry Metabolomics Data, Related to Figure 6

Document S2. Article plus Supplemental Information
