# Supplementary material for: Mapping Interactions of Microbial Metabolites with Human G-Protein-Coupled Receptors
Source: Cell Host Microbe. 2019 Aug 14;26(2):273–282.e7. doi: 10.1016/j.chom.2019.07.002 (PMC6706627; doi:10.1016/j.chom.2019.07.002)
Supplement: Document S1. Figures S1–S11 [file mmc1.pdf]

Cell Host & Microbe, Volume *26*

## Supplemental Information

# Mapping Interactions of Microbial Metabolites

# with Human G-Protein-Coupled Receptors

**Dominic A. Colosimo, Jeffrey A. Kohn, Peter M. Luo, Frank J. Piscotta, Sun M. Han, Amanda J. Pickard, Arka Rao, Justin R. Cross, Louis J. Cohen, and Sean F. Brady**

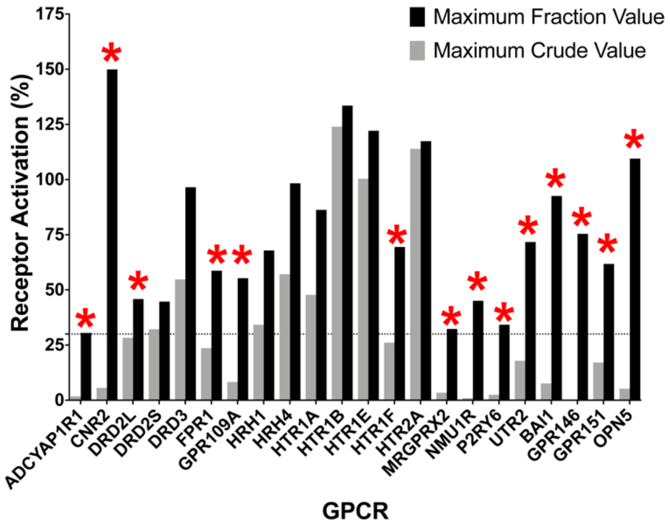


**Figure S1. Comparison of crude extract and fractionated extracts, related to Figure 2.** Activity derived from the crude extract is shown side-by-side with the activity of the maximum active fraction for each of the 22 validated GPCRs. Red asterisks indicate GPCRs that did not reach hit threshold in the original screen.

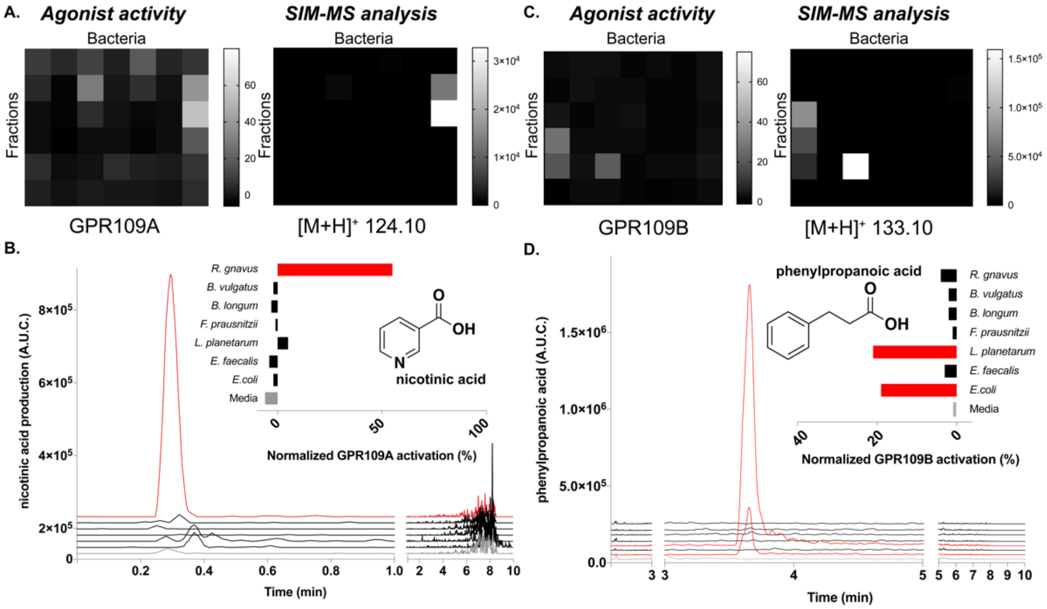


**Figure S2. Heatmaps of GPR109A agonism activity and MS-based detection of nicotinic acid in early, polar fractions 1-6, related to Figure 3.** A. Heatmaps of GPR109A agonism activity and MS-based detection of nicotinic acid in early, polar fractions 1-6. B. GPR109A agonism and SIM-MS analysis of nicotinic acid of fraction 3 across all SIHUMI members. C. Heatmaps of GPR109B agonism activity and MS-based detection of phenylpropanoic acid in early, polar fractions 1-6. D. GPR109B agonism and SIM-MS analysis of phenylpropanoic acid of fraction 3 across all SIHUMI members.

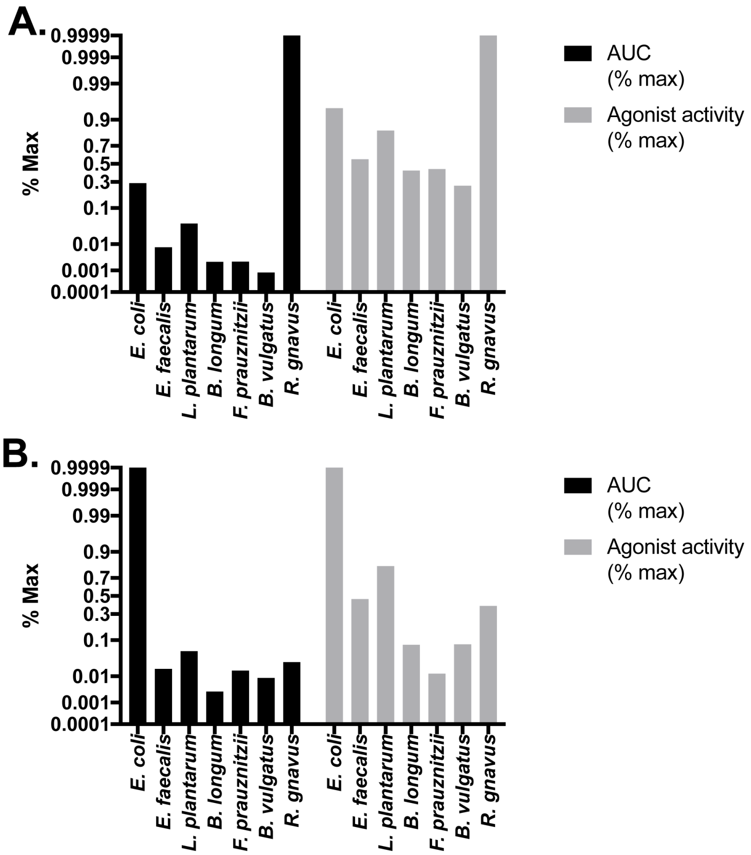


**Figure S3. Correlation of chemical MS-based identification of aromatic amines and neurotransmitter receptor activation in representative fraction from each bacterium, related to Figure 3.** A. Correlation of chemical MS-based identification of tryptamine and serotonin receptor activation in representative fraction from each bacterium. Values represent peak height of ion extractions for the mass of tryptamine and the activation of the serotonin receptor HTR2A normalized to maximum peak height and agonist activity, respectively. B. Correlation of chemical MS-based identification of tyramine and dopamine receptor activation in representative fraction from each bacterium. Values represent peak height of ion extractions for the mass of tyramine and the activation of the dopamine receptor DRD3 normalized to maximum peak height and agonist activity, respectively.

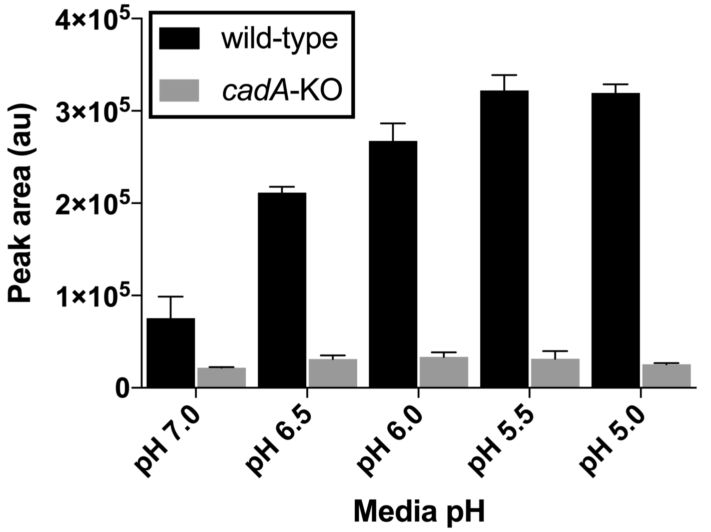


**Figure S4. pH-dependent production of cadaverine by *E. coli* LF82, related to Figure 4.** High-resolution mass spectrometry analyses of *E. coli* LF82 cultures exposed to various pH levels demonstrate increase in cadaverine production in response to decrease in the media pH and dependence of this effect on *cadA* gene. Error bars are SD, n=2.

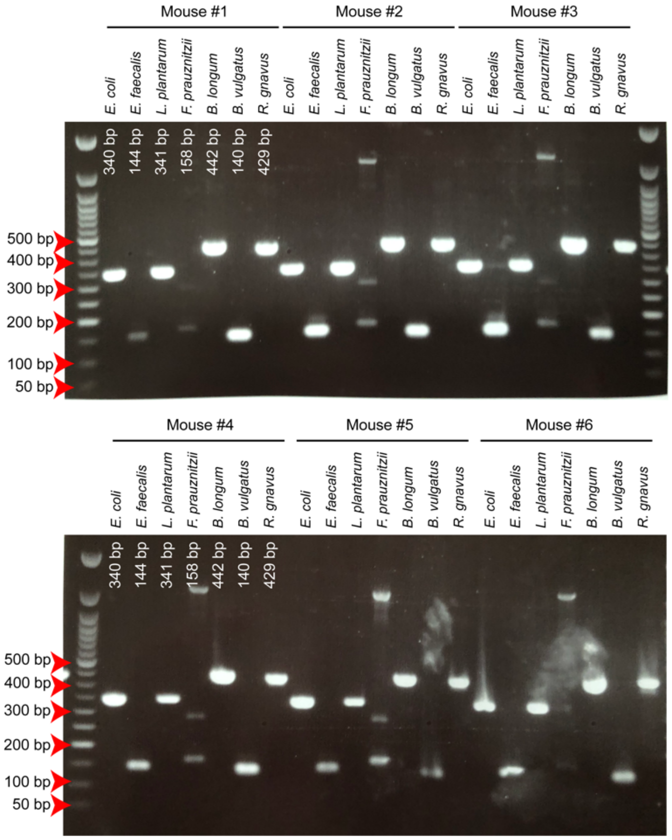


**Figure S5. PCR analysis of each SIHUMI strain in the stool of consortium-gavaged mice, related to STAR Methods.** Species-specific primers targeting variable region of 16s rRNA used in PCR analysis to identify presence of SIHUMI members in stool of gavaged mice ten days after inoculation.

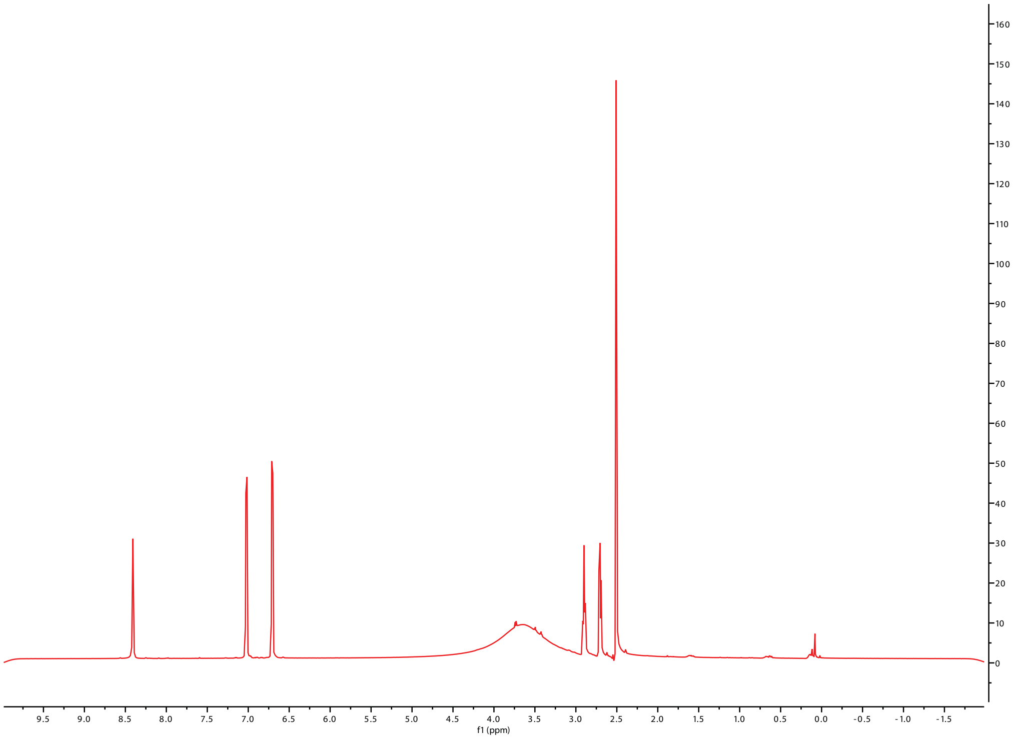


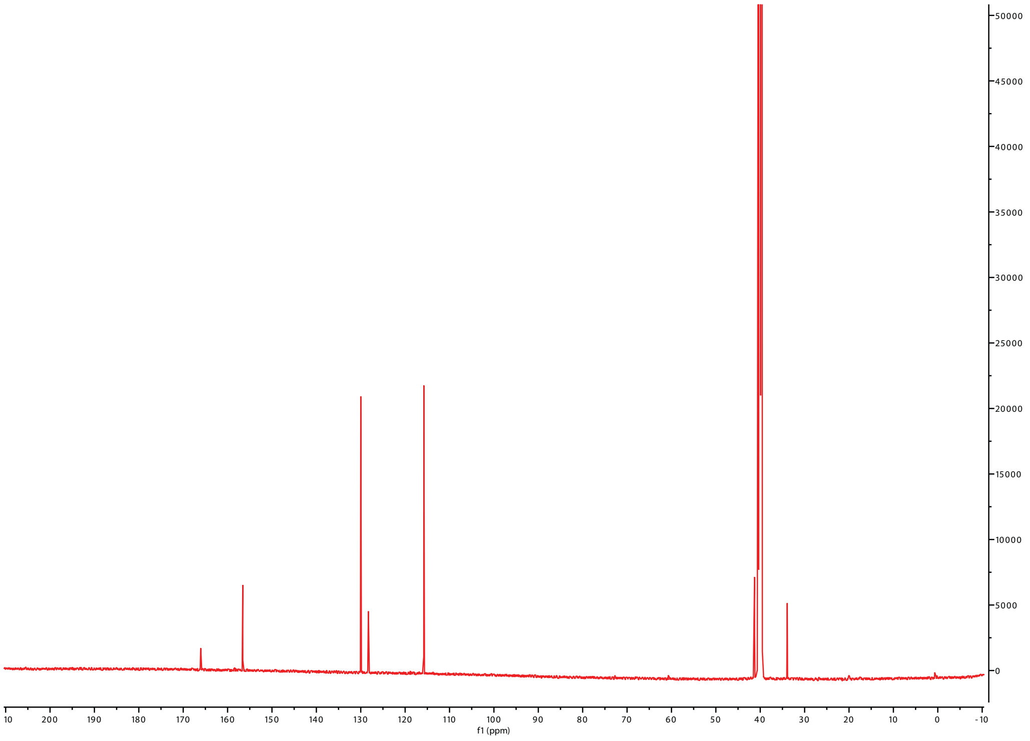


**Figure S6. $^1$H and $^{13}$C NMR data for isolated tyramine (DMSO-$d_6$), related to STAR Methods.**

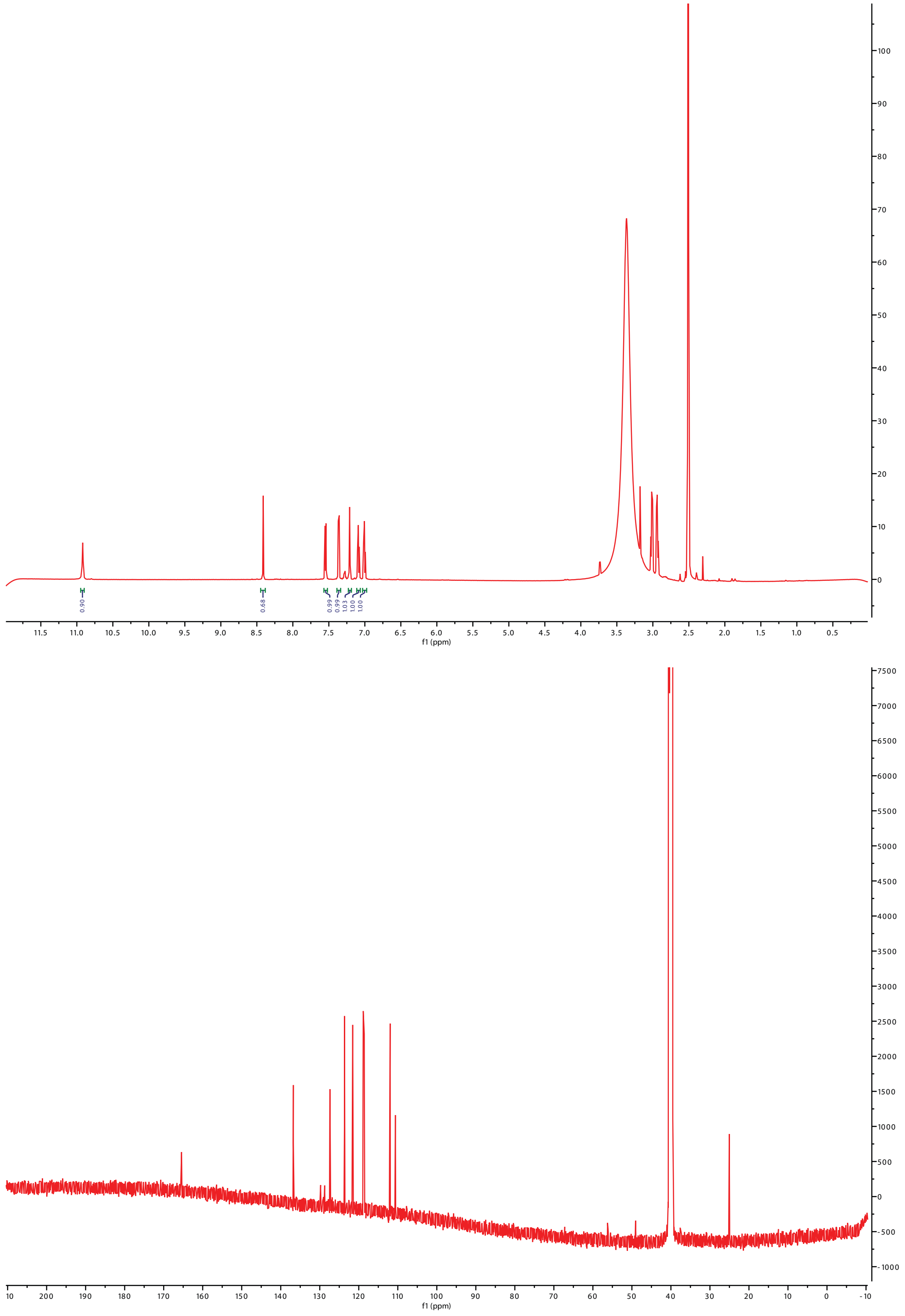


**Figure S7. $^{1}$H and $^{13}$C NMR data for isolated tryptamine (DMSO-$d_6$), related to STAR Methods.**

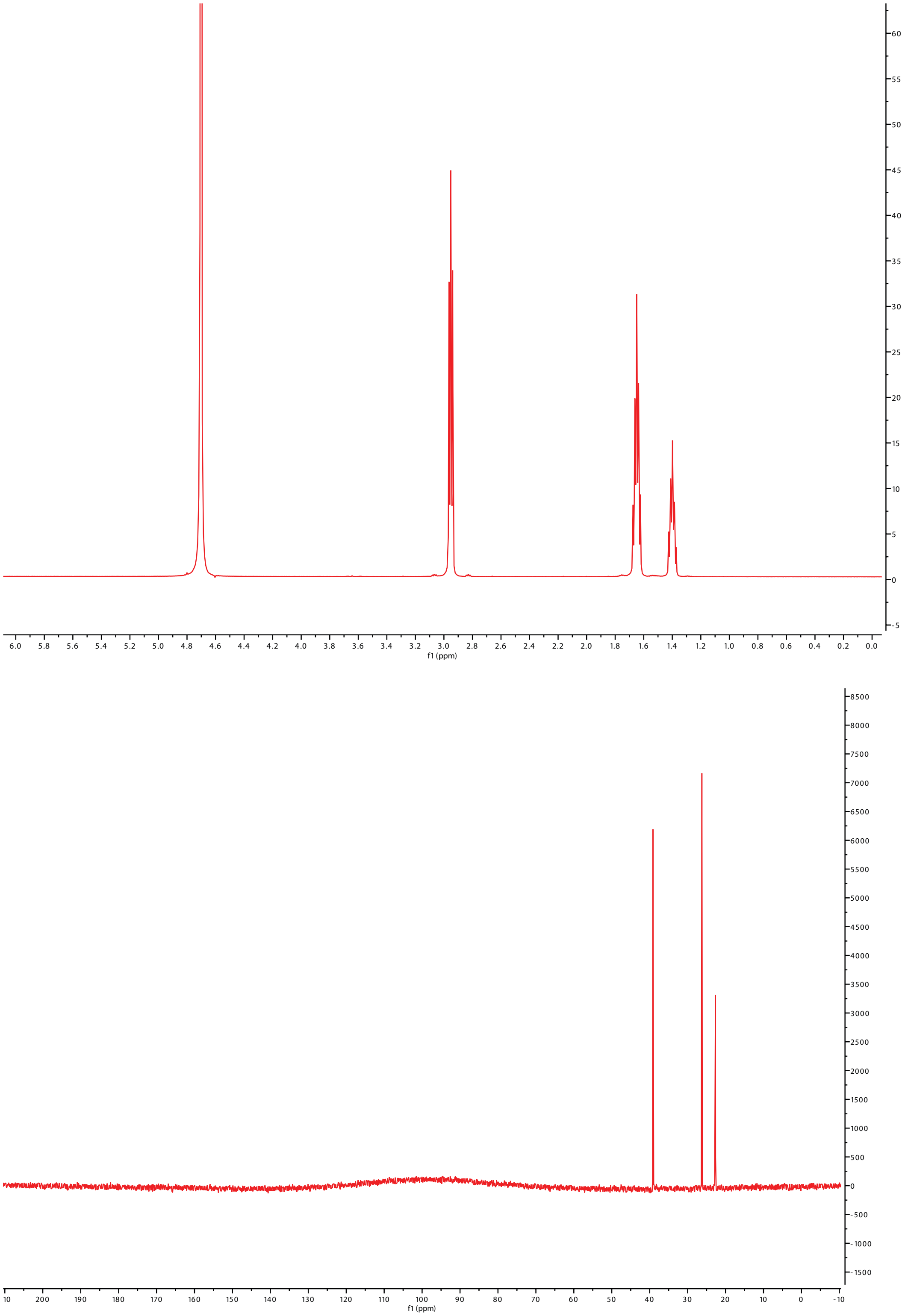


**Figure S8. $^{1}H$ and $^{13}C$ NMR data for isolated cadaverine ($D_2O$), related to STAR Methods.**

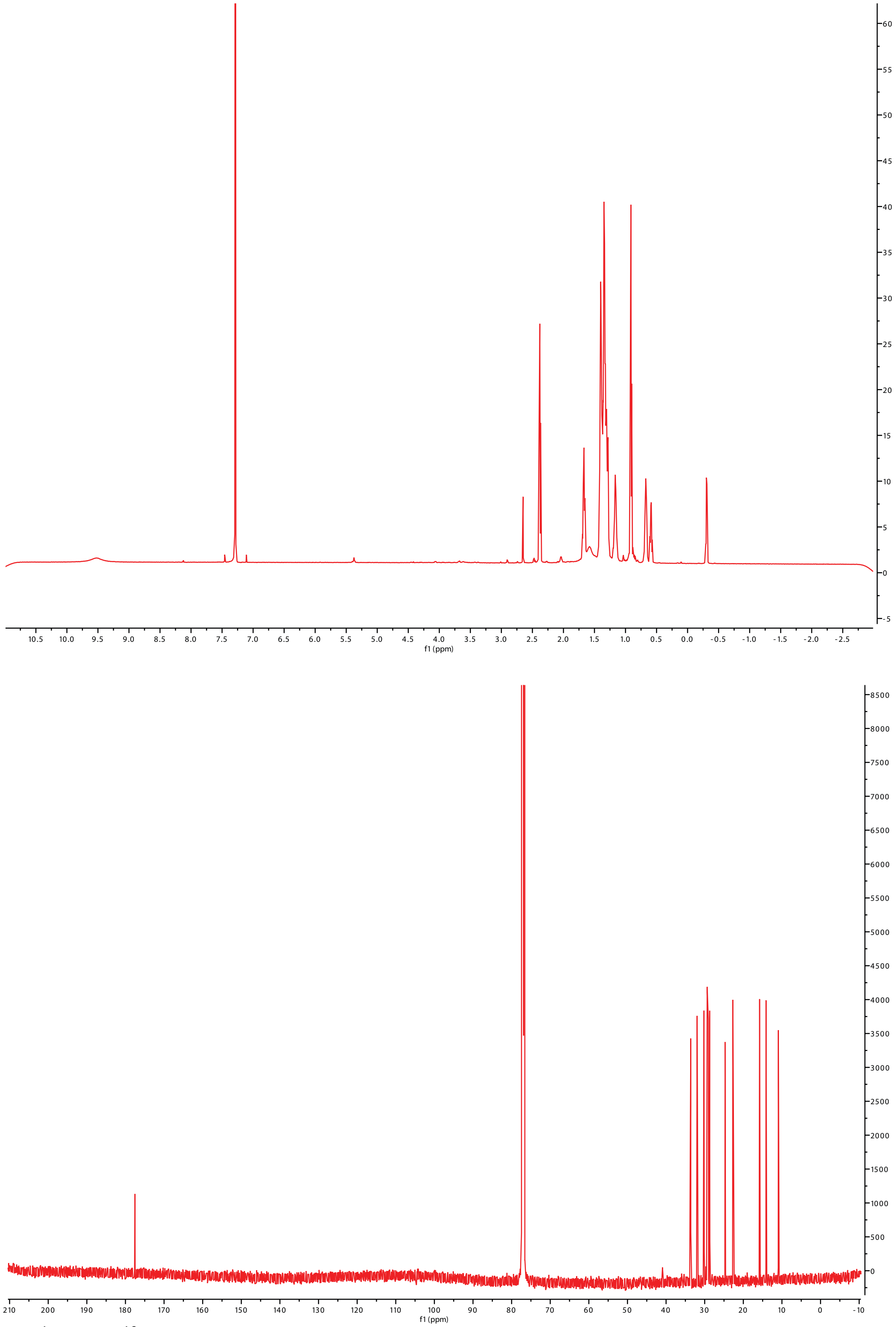


**Figure S9. $^{1}H$ and $^{13}C$ NMR data for isolated 9,10-methylenehexadecanoic acid ($CDCl_3$), related to STAR Methods.**

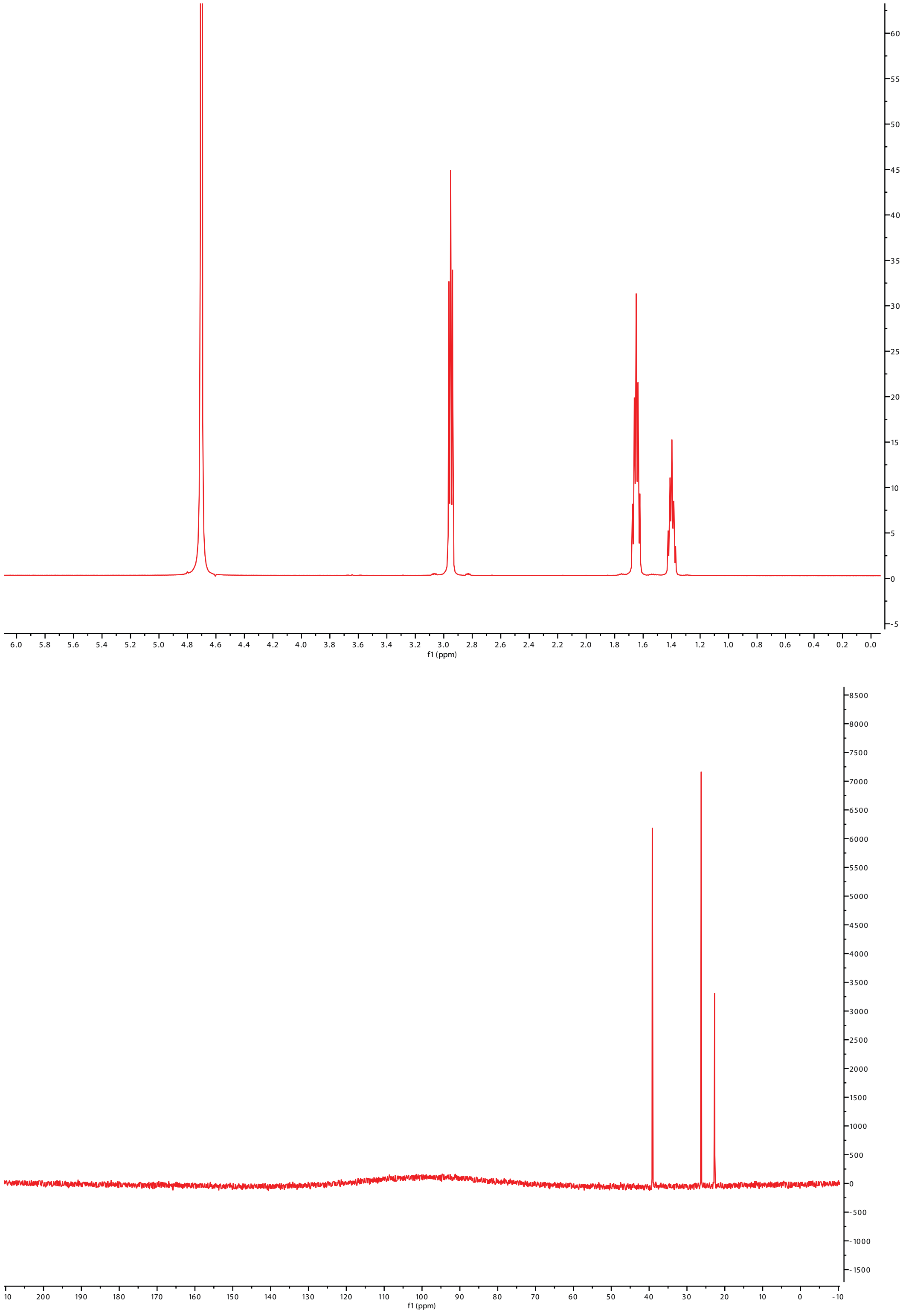


**Figure S10. $^{1}$H and $^{13}$C NMR data for isolated 12-methyltetradecannoic acid ($CDCl_3$), related to STAR Methods.**

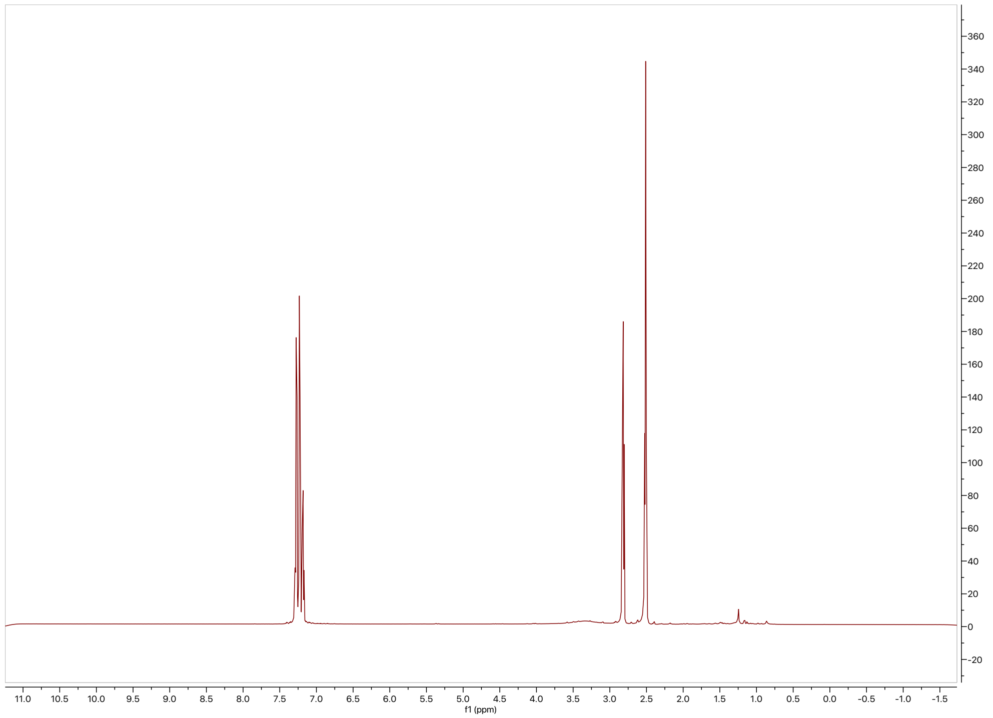


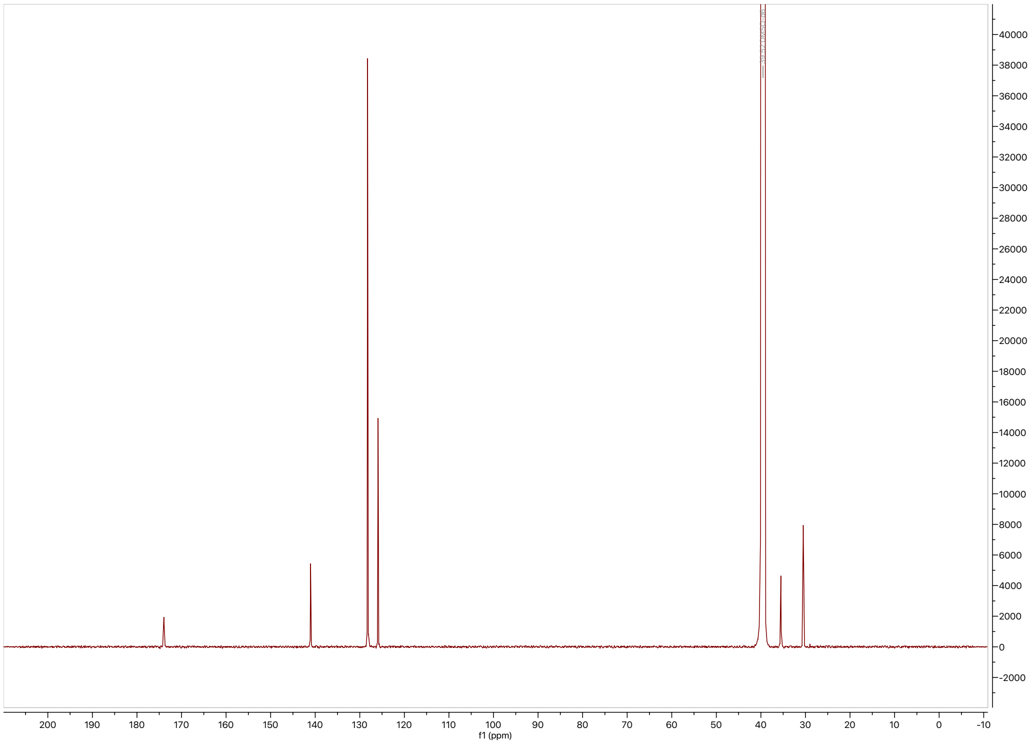


**Figure S11. $^1$H and $^{13}$C NMR data for isolated phenylpropanoic acid (DMSO-$d_6$), related to STAR Methods.**
